# Acupuncture as treatment of cancer-therapy induced fatigue: a critical systematic review with a focus on the methodological assessment of blinding

**DOI:** 10.1007/s00432-025-06395-4

**Published:** 2026-01-08

**Authors:** Samuel Voigtländer, Jennifer Dörfler, Jutta Hübner

**Affiliations:** Klinik für Innere Medizin II, Hämatologie und Internistische Onkologie, Universitätsklinikum Jena, Am Klinikum 1, 07747 Jena, Germany

**Keywords:** Cancer-related fatigue, Acupuncture, Integrative oncology, Systematic review, Randomized controlled trials, Blinding

## Abstract

**Background:**

Acupuncture is a method of traditional Chinese medicine that has been adapted in the Western world. The objective of this study was to critically assess the evidence presented in randomized controlled trials (RCTs) about the effectiveness of acupuncture on fatigue in cancer patients.

**Method:**

In April 2024 a systematic search was conducted searching five electronic databases to find studies concerning the use, effectiveness and potential harm of acupuncture therapy on cancer patients.

**Results:**

From all (1599) search results, 15 studies with 1346 patients were included. Acupuncture methods varied (e.g., traditional-, electro-, mind-regulating and ATAS-acupuncture) and were compared to sham acupuncture, usual care, or other controls. Studies comparing acupuncture to sham acupuncture reported mixed results: while some found significant effects on cancer-related fatigue, others found no advantages. Studies comparing acupuncture to usual care or waitlist controls often reported positive effects. However, the reliability of these findings is limited, as 14 of 15 studies were rated as “high risk of bias” by the RoB-2 tool due to issues like insufficient blinding and incomplete data analysis. Only one study, with low risk of bias, showed a significant reduction in fatigue with acupuncture compared to sham acupuncture (*p* < 0.001). GRADE evaluation also showed very low certainty of evidence.

**Conclusion:**

The heterogenous results and methodological limitations of the existing studies prevent us from drawing definitive conclusions about the effectiveness of acupuncture in the treatment of cancer-related fatigue. Despite the inclusion of 15 studies, the overall evidence remains insufficient due to widespread problems in study design and inconsistent results. This analysis highlights the need to use more rigorous designs and more comprehensive assessment tools in future studies to better understand the role of acupuncture in the management of fatigue after cancer treatment.

**Supplementary Information:**

The online version contains supplementary material available at 10.1007/s00432-025-06395-4.

## Introduction

Cancer-Related Fatigue (CRF) is a common and debilitating symptom in patients undergoing oncology therapies and significantly affects their quality of life (Choi et al. 2022). Conventional pharmacological treatments for CRF have been shown to have limited efficacy, so complementary and alternative medicine (CAM) approaches, including acupuncture, are increasingly being used (Lau et al. 2016). Acupuncture, a key component of traditional Chinese medicine, involves the insertion of fine needles into specific points on the body to regulate physiological functions. Although numerous studies have demonstrated the effectiveness of acupuncture in the treatment of CRF, the credibility of these studies is often questioned due to potential bias and methodological limitations.

Despite the controversy over the effectiveness of acupuncture, clinical practice guidelines recommend the evidence-based use of integrative therapies. For example, guidelines by Greenlee et al. (2017) recommend acupuncture as a viable option for the treatment of CRF during and after breast cancer treatment, emphasizing the potential benefits of acupuncture and the relatively low risk of side effects compared to pharmacological treatments.

Several systematic reviews and meta-analyses have evaluated the role of acupuncture in the treatment of CRF. For example, Yuanqing et al. (2020) concluded that acupuncture and moxibustion can effectively reduce the degree of fatigue in cancer patients, with true acupuncture showing greater benefits than sham acupuncture. In addition, studies have shown that acupuncture in conjunction with routine treatment improves both physical and mental fatigue, anxiety, depression and overall quality of life (Tan et al. 2021). However, the subjective nature of CRF and the placebo effect must be carefully considered when interpreting these results (Zhao et al. 2020).

One of the biggest challenges in acupuncture research is the difficulty in performing adequate blinding. The “de qi” sensation, a mixture of numbness, tingling or a dull ache that is considered an indicator of effective needle placement, complicates the blinding process, as is difficult to reproduce or is completely neglected in placebo-controlled studies, making it possible for the patients to distinguish between real and sham acupuncture (Li and Liu 2021). Additionally, achieving the “de qi” sensation relies heavily on the dynamic interaction between the practitioner and the patient. This interaction further distinguishes the context of real acupuncture from sham procedures.

The aim of this systematic review is to critically evaluate the biases in the existing studies of acupuncture for CRF in order to gain a clearer understanding of the true effectiveness of acupuncture and to inform the design of future research. By identifying and addressing these biases, future studies can be conducted more accurately, ultimately leading to more reliable conclusions and better patient care.

## Method

### Criteria for inclusion and exclusion

Inclusion and exclusion criteria are listed in Table 1 based on a PICO-model. Only randomized controlled trials were included if they reported patient-relevant outcomes after treatment of adult cancer patients with any intervention containing acupuncture. Because of the wide range of application fields, all cancer entities were included. Language restrictions were made to English and German.

**Table 1:** Search strategy

Database	Searchstring
Ovid Medline	**1** (acupunct* or acupoints* or electroacupunct* or electro-acupunct* or auriculoacupunct* or ear-acupunct*).ti,ab,kw. or Acupuncture/ or Acupuncture Therapy/ **2** exp Fatigue/ or (fatigue$ or tired$ or sleepy or sleepi$ or drows$ or lassitude or lethargy$ or weary or weariness or exhaustion or exhausted or lacklustre or apathy or apathetic or ((asthenia or asthenic) adj3 syndrome) or ((lack or loss or lost) adj3 (energy or vigour or vigor)) or (feeling adj3 (drained or sleepy or sluggish or weak$))).mp **3** exp neoplasms/ or neoplasm$.mp or cancer$.mp. or tumo?r$.mp. or malignan$.mp. or oncolog$.mp. or carcinom$.mp. or leuk?emia.mp. or lymphom$.mp. or sarcom$.mp. or chemo$.mp **4** 1 AND 2 AND 3 **5** (4 and humans/) or (4 not animals/) **6** ((((comprehensive* or integrative or systematic*) adj3 (bibliographic* or review* or literature)) or (meta-analy* or metaanaly* or “research synthesis” or ((information or data) adj3 synthesis) or (data adj2 extract*))).ti,ab. or (cinahl or (cochrane adj3 trial*) or embase or medline or psyclit or (psycinfo not “psycinfo database”) or pubmed or scopus or “sociological abstracts” or “web of science” or central).ab. or (“cochrane database of systematic reviews” or evidence report technology assessment or evidence report technology assessment summary).jn. or Evidence Report: Technology Assessment*.jn. or (network adj1 analy*).ti,ab.) or (((review adj5 (rationale or evidence)).ti,ab. and review.pt.) or meta-analysis as topic/ or Meta-Analysis.pt.) **7** 5 AND 6
Cochrane	**#1** (acupunct* or acupoints* or electroacupunct* or electro-acupunct* or auriculoacupunct* or ear-acupunct*):ti,ab,kw or [mh acupuncture] or [mh “acupuncture therapy”] **#**2 [mh Fatigue] or (fatigue* or tired* or sleepy or sleepi* or drows* or lassitude or letharg* or weary or weariness or exhaustion or exhausted or lacklustre or ((asthenia or asthenic) near/3 syndrome) or ((lack or loss or lost) near/3 (energy or vigour or vigor)) or apathy or apathetic or lassitude or weakness or lethargy or lethargic or (feeling near/3 (drained or sleepy or sluggish))):ti,ab,kw **#3** [mh neoplasms] or neoplasm* or cancer? or tum*r? or malignan* or oncolog* or carcinom* or leuk*mia or lymphoma? or sarcoma? **#4 #**1 AND #2 AND #3
Ebsco-PsychINFO	**S1** TI (acupunct* or acupoints* or electroacupunct* or electro-acupunct* or auriculoacupunct* or ear-acupunct*) or AB (acupunct* or acupoints* or electroacupunct* or electro-acupunct* or auriculoacupunct* or ear-acupunct*) or MH (acupuncture or “acupuncture therapy”) **S2** ((DE “Neoplasms” OR DE “Benign Neoplasms” OR DE “Breast Neoplasms” OR DE “Endocrine Neoplasms” OR DE “Leukemias” OR DE “Melanoma” OR DE “Metastasis” OR DE “Nervous System Neoplasms” OR DE “Terminal Cancer”) OR (TX neoplasm* OR TX cancer OR TX tumo#r OR TX malignan* OR DE „oncology “ OR TX oncolog* OR TX carcinom* OR TX leuk#emia OR TX lymphoma OR TX sarcoma)) **S3** DE “Fatigue” or TX (fatigue* or tired* or sleepy or sleepi* or drows* or lassitude or letharg* or weary or weariness or exhaustion or exhausted or lacklustre or ((asthenia or asthenic) N3 syndrome) or ((lack or loss or lost) N3 (energy or vigour))) or TX (apathy or apathetic or lassitude or weakness or lethargy or lethargic or (feeling N3 (drained or sleepy or sluggish))) **S4** S1 AND S2 AND S3 **S5** ((comprehensive* OR integrative OR systematic*) N3 (bibliographic* OR review* OR literature)) OR (meta-analy* or metaanaly* or “research synthesis” OR ((information OR data) N3 synthesis) OR (data N2 extract*)) OR ((review N5 (rationale OR evidence)) AND DE “Literature Review”) OR (AB(cinahl OR (cochrane N3 trial*) OR embase OR medline OR psyclit OR pubmed OR scopus OR "sociological abstracts” OR “web of science” OR central)) OR DE “Meta Analysis” OR (network N1 analy*) **S6** S4 AND S5
OVID Embase	**1** (acupunct* or acupoints* or electroacupunct* or electro-acupunct* or auriculoacupunct* or ear-acupunct*).ti,ab,kw. or Acupuncture/ or Acupuncture Therapy/ **2** exp cancer fatigue/ or exp fatigue/ or (fatigue$).ti,ab,kw or (tired$ or weary or weariness or exhaustion or exhausted or sleepy or sleepi$ or drows$ or lacklustre or astheni$ or asthenia$ or ((asthenia or asthenic) adj3 syndrome) or ((lack$ or loss or lost) adj2 (energy or vigour or vigor)) or apathy or apathetic or lassitude or letharg$ or (feeling adj3 (drained or sleepy or sluggish or weak$))).mp **3** exp neoplasms/ or neoplasm$.mp or cancer$.mp. or tumo?r$.mp. or malignan$.mp. or oncolog$.mp. or carcinom$.mp. or leuk?emia.mp. or lymphom$.mp. or sarcom$.mp **4** 1 AND 2 AND 3 **5** (4 and humans/) or (4 not animals/) **6** ((((comprehensive* or integrative or systematic*) adj3 (bibliographic* or review* or literature)) or (meta-analy* or metaanaly* or “research synthesis” or ((information or data) adj3 synthesis) or (data adj2 extract*))).ti,ab. or (cinahl or (cochrane adj3 trial*) or embase or medline or psyclit or (psycinfo not “psycinfo database”) or pubmed or scopus or “sociological abstracts” or “web of science” or central).ab. or (“cochrane database of systematic reviews” or evidence report technology assessment or evidence report technology assessment summary).jn. or Evidence Report: Technology Assessment*.jn. or (network adj1 analy*).ti,ab.) or (exp Meta Analysis/ or ((data extraction.ab. or selection criteria.ab.) and review.pt.)) **7** 5 AND 6
Ebsco-CINAHL	**S1** TI (acupunct* or acupoints* or electroacupunct* or electro-acupunct* or auriculoacupunct* or ear-acupunct*) or AB (acupunct* or acupoints* or electroacupunct* or electro-acupunct* or auriculoacupunct* or ear-acupunct*) or MH (acupuncture or “acupuncture therapy”) **S2** (MH “Cancer Fatigue”) or TX ((lack N3 energy) or (loss N3 energy) or (lost N3 energy) or (lack N3 vigo#r) or (loss N3 vigo#r) or (lost N3 vigo#r) or (feeling adj3 (drained or sleepy or sluggish or weak$)) or (fatigue* or tired* or sleepy or sleepi* or drows* or lassitude or letharg* or weary or weariness or exhaustion or exhausted or lacklustre or (asthenia N3 syndrome) or ((asthenic or asthenia) N3 syndrome)) **S3** (MH “Neoplasms+” OR TX neoplasm* OR TX cancer OR TX tumo#r OR TX malignan* OR TX oncolog* OR TX carcinom* OR TX leuk#emia OR TX lymphoma OR TX sarcoma) **S4** S1 AND S2 AND S3 **S5** (TI (systematic* n3 review*)) or (AB (systematic* n3 review*)) or (TI (systematic* n3 bibliographic*)) or (AB (systematic* n3 bibliographic*)) or (TI (systematic* n3 literature)) or (AB (systematic* n3 literature)) or (TI (comprehensive* n3 literature)) or (AB (comprehensive* n3 literature)) or (TI (comprehensive* n3 bibliographic*)) or (AB (comprehensive* n3 bibliographic*)) or (TI (integrative n3 review)) or (AB (integrative n3 review)) or (JN “Cochrane Database of Systematic Reviews”) or (TI (information n2 synthesis)) or (TI (data n2 synthesis)) or (AB (information n2 synthesis)) or (AB (data n2 synthesis)) or (TI (data n2 extract*)) or (AB (data n2 extract*)) or (TI (medline or pubmed or psyclit or cinahl or (psycinfo not “psycinfo database”) or “web of science” or scopus or embase)) or (AB (medline or pubmed or psyclit or cinahl or (psycinfo not “psycinfo database”) or “web of science” or scopus or embase or central)) or (MH “Systematic Review”) or (MH “Meta Analysis”) or (TI (meta-analy* or metaanaly*)) or (AB (meta-analy* or metaanaly*)) or network n1 analy* **S6** S4 AND S5

### Study selection

**Fig. 1:**
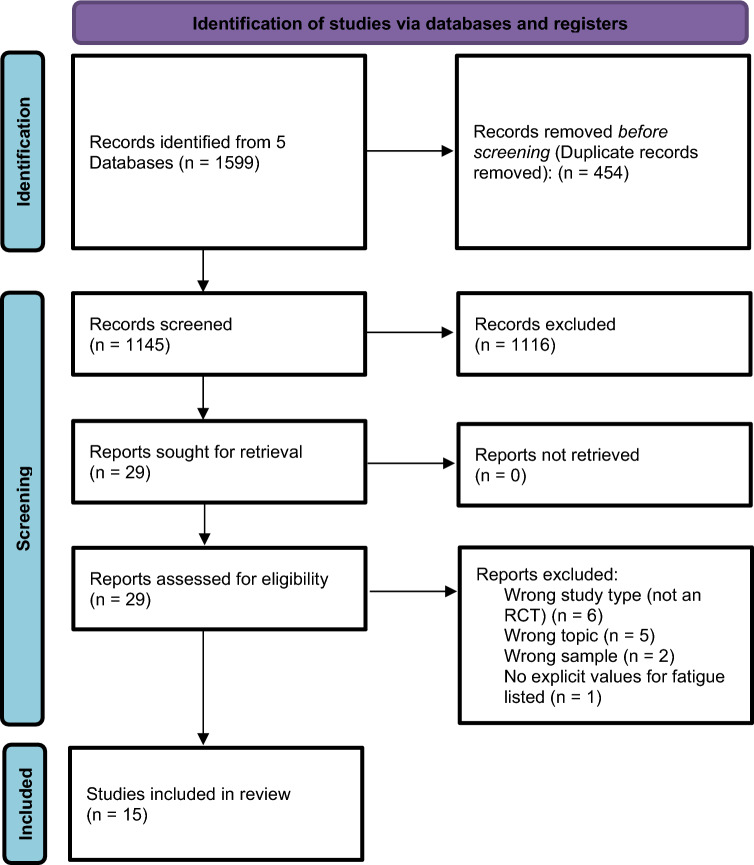
Consort diagram.* RCT* Randomized controlled trial

A systematic research was conducted using five databases (Medline (Ovid), CINAHL (EBSCO), EMBASE (Ovid), Cochrane CENTRAL and PsycINFO (EBSCO)) in April 2024. For each of these databases a complex search strategy was developed consisting of a combination of Mesh Terms, keywords and text words in different spellings connected to cancer and acupuncture therapy (Fig. 1). After importing the search results into EndNote 20, all duplicates were removed and a title–abstract–screening was carried out by two independent reviewers (JD, SV). In case of disagreement, a third reviewer was consulted (JH) and consensus was made by discussion. After that all the full texts were retrieved and screened again independently by both reviewers (JD, SV). Additionally, bibliography lists of all retrieved articles were searched for relevant studies.

### Assessment of risk of bias and methodological quality

All characteristics were assessed by two independent reviewers (JD, SV). In case of disagreement a third reviewer was consulted (JH) and consensus was made by discussion. The risk of bias in the included studies was analyzed with the Cochrane revised Risk of Bias Tool 2.0 (Eldridge et al. 2016) and additionally the GRADE evaluation system (Table 6). Additional criteria concerning methodology were application of power analysis and adequacy of statistical tests (e.g. control of premises or multiple testing). A special focus was placed on possible influences on blinding in regard to the inability of reproducing certain effects like electrical stimulation or the de qui sensation and therefor giving feedback on those and the interaction with the acupuncturist, which is particularly important in studies comparing real acupuncture (RA) with sham acupuncture (SA).

### Data extraction

Data extraction was performed by one reviewer (SV) and controlled by two independent reviewers (JD/ JH). As a template for data extraction, the evidence tables from the national Guideline on Complementary and Alternative Medicine in Oncological Patients of the German Guideline Program in Oncology (Wissenschaftlichen et al. 2024) were used. Concerning systematic reviews, only data from primary literature meeting the inclusion criteria of the present work were extracted.

## Results

### Characteristics of included studies

The systematic research revealed 1599 results. At first, duplicates were removed leaving 1145 studies. After screening title and abstract, 29 studies remained for complete review. Finally, 15 publications were analyzed in this review (consort diagram, Fig. 1).

Concerning all relevant studies, 1346 patients were included and 1138 of them were analyzed, due to 208 dropouts (Table 2). The age of patients ranged from 18 to 80 years with a range of age from 29 to 62 years. 1207 participants were female and 137 were male.

**Table 2:** Inclusion and exclusion criteria

PICO	Inclusion criteria	Exclusion criteria
Patient	Cancer patients all entities and stages) Adult patients (age > 18)	Patients with only precancerous conditions or Carcinoma in situ Preclinical studies Study population with more than 20% children or precancerous conditions, patients with a diagnosis of insomnia
Intervention	Different types of traditional acupuncture and electroacupuncture	Combinations with other active interventions such as Moxibustion
Comparison	All possible control groups (placebo, standard care, observation, sham group)	Other study types (one-armed/non-controlled studies, case report or series)
Outcome	Fatigue as a primary or secondary endpoint or as a subpoint of a higher-level score such as quality of life, etc.	Fatigue as part of Insomnia
Study types	Only RCTs, full publications published in Journals with peer-review	Meta-analyses, systematic reviews, Grey literature (conference articles, abstracts, letters, ongoing studies, unpublished literature
Language	German and English	

The 15 studies included were published between 2007 and 2020. The method of acupuncture differs in the studies; traditional acupuncture was used in 11 (Cheng et al. 2017; Deng et al. 2013; Deng et al. 2018; Du et al. 2021; Li et al. 2020; Li et al. 2023a; Eonju et al. 2019; Molassiotis et al. 2012; Molassiotis et al. 2013; Molassiotis et al. 2007; Smith et al. 2013) and electroacupuncture in 2 studies (Hou et al. 2017; Mao et al. 2014). In 2 studies (Lu et al. 2012; Balk et al. 2009) electrostimulation of the acupuncture points was only partially applied. In addition, different points were chosen for acupuncture. Regarding interventions and controls, in 5 trials (Cheng et al. 2017; Deng et al. 2013; Deng et al. 2018; Lu et al. 2012; Balk et al. 2009) acupuncture was compared with sham acupuncture (in one of them with an additional heat lamp) (Balk et al. 2009), in another one mind regulating acupuncture was applied and both groups got additional treatment with Shenqi Fuzheng (intramuscular or intravenous herbal injections) and consultations (Li et al. 2023a), in 4 studies (Du et al. 2021; Li et al. 2023a; Eonju et al. 2019; Molassiotis et al. 2012) acupuncture (one with Korean Saam acupuncture) was compared to control groups which just got the usual care. In 4 studies (Li et al. 2020; Smith et al. 2013; Hou et al. 2017; Mao et al. 2014) acupuncture (two of them with electrostimulation (Hou et al. 2017; Mao et al. 2014), one of them with ATAS, acupuncture (Li et al. 2020)) was compared to sham acupuncture and a third control group which just got usual care. One study (Molassiotis et al. 2013) compared acupuncture to self-acupuncture and a control group, one (Molassiotis et al. 2007) compared acupuncture to acupressure and sham acupressure. Six studies actively tried to achieve a “de qui” sensation by stimulating the needles manually (Cheng et al. 2017; Deng et al. 2013; Eonju et al. 2019; Smith et al. 2013; Mao et al. 2014; Lu et al. 2012), in one study, it was only explained that it could be experience in order not to jeopardize the blinding (Balk et al. 2009). Detailed characterization of the included studies may be seen in Table 3.

**Table 3:** Short evidence table

References	Study type	n/Cancer type/Drop out	Intervention/Duration	Endpoints	Outcomes
Balk et al. (2009)	RCT	Randomized (A/B): n = 27 (16/11) Drop-outs (A/B): 4 (1/0(during acupuncture)+0/3(no assesments)) Assessed (A/B): n = 23 (15/8) NSCLC diagnosis	A: MA+Low-frequency electrical stimulation (Ki-3(−); St-36(+))+heat lamp (CV-6) B: SA (with Park Sham Device, a blunt needle, electrical simulation off, heat lamp on low) 6–4 weeks, 2× per week for 30 min	T0: Baseline T1: Week 3 T2: Week 6 T3: Week 10 1. CRF 2. Form-36 (SF-36) 3.Cancer related fatigue distress scale (CRFDS)	1. T0, mean/median: A: 31.9/31, B: 36.6/38; Repeated Measures ANOVA: treatment *p* = 0.457, time *p* = 0.106 2. Repeated Measures ANOVA PCS-Score: treatment *p* = 0.972, time p = 0.370; MCS-Score treatment *p* = 0.197, time *p* = 0.454; ns. for change within or comparison between groups at any time point; comparison difference at T0 between the groups over all time points, group effect *p *= 0.457, time effect *p* = 0.106, ns 3. Repeated Measures ANOVA, mean CESD Scores: treatment *p* = 0.294, time *p *< 0.009; ns. for change within or between groups at any time point, group effect *p* = 0.972, time effect *p* = 0.370, ns
Cheng et al. (2017)	RCT	Randomized (A/B): n = 28 (14/14) Drop-outs: 0 Assessed (A/B): n = 28 (14/14) NSCLC diagnosis	A: MA B: SA (Sham acupuncture needles, no further information) 4 weeks; 2×/week	T0: Baseline (week -2) T1: Week 0 T2: Week 2 T3: Week 4 1. Fatigue (BFI-C) 2. Quality of life (FACT-LCS)	1. T0, mean (SD): 6.2(A); 6.6(B) *p* = 0.35; T1: 5.1(A); 6.3(B) *p* < 0.01; T2: 5.2(A); 6.6(B) *p* = 0.005; T3: 4.5(A); 7.1(B) *p* < 0.001 2. T0, mean (SD): 86.4(A); 82.1(B) *p* = 0.23; T1: 93.8(A); 88.5(B) *p* = 0.11; T2: 96.1(A); 90.3(B) *p* = 0.04; T3: 98.0(A); 89.3(B) *p *= 0.002; All results significant in favor of acupuncture
Deng et al. (2013)	RCT	Randomized (A/B): n = 101 (49/52) Drop-outs (A/B): 27 (2/1 (before acupuncture)+13/11) Assessed (A/B): n = 74 (34/40) Mixed cancer diagnoses	A: MA+UC B: SA (Sham acupuncture needles (blunt) and points that are a few millimeters away from the meridian points of true acupuncture)+UC 6 weeks; 1×/week	Baseline T0: Means of day-14 and -7 Post-treatment difference T1: Means of day 42 and 49 1. Fatigue (BFI) 2. Quality of life (FACT-G, HADS)	Post-treatment difference, SD adjusted for baseline scores: 1. Between the groups: 0.04 (95%CI − 0.57, 0.66) *p* = 0.9; ns 2. Between the groups: FACT-G (overall): 0.10 (95%CI − 3.37, 3.57) *p* = 1.0; ns.; HADS: Depression: − 0.10 (95%CI − 1.61, 1.41) *p* = 0.9; ns.; Anxiety: − 0.21 (95%CI − 1.35, 0.93) *p* = 0.7; ns
Du et al. (2021)	RCT	Randomized (A/B): n = 61 (30/31) Drop-out (A/B): 11 (4/7) Assessed (A/B): n = 50 (26/24) Mixed cancer diagnoses	A: MA+Ctx B: Just Ctx Ctx: 2 times 3 weeks Acupuncture on day-1,0,1,2 of each cycle (8 times in total)	T0: Baseline T1: 6 weeks after therapy start (3 weeks after last acupuncture) 1. PFS 2. EORTC-QLQ-C30	1. A versus B behavior, emotions, perception, cognition sub scores: T0: *p* = 0.489, *p* = 0.859, *p* = 0.276, *p* = 0.050, *p* = 0.240 ns.; T1: perception: *p* = 0.019 -> sign. lower for A; Rest: *p* = 0.095, *p* = 0.243, *p* = 0.908, *p* = 0.096; ns T0 versus T1: Arm A: *p* < 0.001, *p* = 0.026, *p* < 0.001, *p *= 0.029, *p* < 0.001; all sign. reduced (favors A); Arm B: *p* = 0.209, *p* = 0.130, *p* = 0.746, *p* = 0.798, *p* = 0.399; ns
					2. A versus B T0: emotional: *p* = 0.015 s. (no comparable baseline) Rest: *p* = 0.950, *p* = 0.133, *p* = 0.577, *p* = 0.761 -> ns T1: physical function: *p* = 0.026 sign. Higher for A Gen. health symptom: *p* = 0.013, *p* = 0.004, *p* = 0.011; sign. reduced for A; Rest: *p* = 0.966, *p* = 0.408, *p* = 0.392; ns., favors A
					T0 versus T1: Arm A: physical, role and social functions: *p* = 0.000, *p* = 0.001, *p* = 0.003; sign. improvement; Cognitive function: *p* = 0.339; ns.; Gen. health, tiredness, dyspnea: *p* = 0.003, *p* = 0.000, *p* = 0.034; sign. improvement Arm B: *p* = 0.571, *p* = 0.746, *p* = 0.539, *p* = 0.223, *p* = 0.095, *p* = 0.968, *p* = 1.0; ns
Li et al. (2020)	RCT	Randomized (A/B/C): n = 40 (20/10/10) Drop-outs (A/B/C): 3 (2/0/1) Assessed(A/B/C): n = 37 (18/10/9) BRCA diagnosis Stage 1–3	A: ATAS A+Ctx B: SA+Ctx C: Ctx ATAS/Sham: 20 weeks, once a week for 60 min	T0: Baseline (randomization) T1: Week 9–12 T2: Week 6–8 after T1 T3: 1 month post Ctx 1. VAS-F 2. MFI-20	Mean (SD): 1. Decreased by 0.351 and 1.198 in arm A compared to arm B and C, respectively; A versus B/C: *p* = 0.004; sign. comparative improvement from week 7 (start of 3rd cycle Ctx.) 2. T2: B/C = 49.0 ± 12.8/56.8 ± 18.7 (peaked), A = 43.1 ± 18.1 (drop); T3: B/C = decreased, A: almost maintained; Change over time: A: *p* = 0.016 B/C: *p* = 0.028 Note: A tabular overview of results and measurements is missing. The results are difficult to follow due to missing values at specific time points and insufficient information on statistical differences
Li et al. (2023a)	RCT	Randomized (A/B/C): n = 136 (68/68) Drop-outs (A/B): 22 (11/11) Assessed (A/B): n = 114 (57/57) BRCA diagnosis stage 0–3	A: MRA+Advise, ShenqiFuzheng injections B: Advise, ShenqiFuzheng injections MRA: 4 weeks; 5×/week	T0: pre-treatment T1: post-treatment 1. MFI-20	1. Mean (SD): T0: A: 73.05 (± 4.07) B:71.61 (± 4.85) T1: A:40.74 (± 3.54) B: 53.07 (± 4.2) sign. greater improvement in arm A (*p* < 0.05)
Eonju et al. (2019)	RCT	Randomized (A/B): n = 26 (13/13) Drop-outs (A/B): 1 (1/0) Assessed (A/B): n = 25 (12/13) PTC diagnosis	A: MA (Saam acupuncture) B: Control group (no acupuncture) 6 weeks, 2× per week for 25–30 min	T0: Baseline T1: after 12 interventions 1. Fatigue severity scale (FSS) 2. Short Form-36 (SF-36)	1. Mean (SD): A T0: 29.92 (13.111) to T1: 19.50 (10.536) B T0: 29.92 (13.029) to T1: 31.15 (12.877) Change over time sign. different (time*group) *p* = 0.011 A: decreased 91.7%, increased 8.3% B: decreased; 46.2%, no change; 7.6%, increased; 46.2%. 11/12 FSS score A: (91.7%); sign. improved 2. A 74.12 versus B 69.02 ns., *p* = 0.085
Molassiotis et al. (2012)	RCT	Randomized (A/B): n = 302 (227/75) Drop-outs (A/B): 56 (46/10) Assessed (A/B): n = 246 (181/65) Breast cancer diagnosis in stage I to IIIa	A: MA+UC (+Fatigue Intervention Brochure) B: UC (+Fatigue Intervention Brochure) 6 weeks, once a week for 20 min	T0: Baseline T1: After 6 weeks 1. Fatigue (MFI) 2. Quality of life (tumor-specific) (FACT-B)	1. Conservative sensitivity analysis for dropouts: effect of A reduced: − 2.49(95%CI, − 3.29 to − 1.69); still sign. (*p* < 0,001) T1: sign. difference between A and B (*p* < 0.001), in favor of A 2. Acupuncture+UC versus UC; post intervention: Sign. group differences in favor of acupuncture (*p* < 0.05)
Molassiotis et al. (2013)	RCT	Randomized (A/B/C): n = 197 (65/67/65) Drop-outs (A/B/C): 46 (9/21/16) Assessed (A/B/C): n = 151 (56/46/49) Breast cancer diagnosis in stage I, II or IIIa	A: MA (bilateral, from a trained acupuncturist) B: MA (bilateral, self-needling after training) C: Control group 20-min session per week for another 4 weeks	T0: Baseline (time of re-randomization = after 6 weeks of the original study) T1: 4 weeks after T0 T2: 12 weeks after T0 1. Fatigue MFI-20 (GF 6–GF 10) to T1 2. endpoints at T2	1. T0-T1 mean difference: A: 0.57 (− 0.18 to 0.04) versus B: 0.54 (− 0.21 to 0.13), ns.; *p* = 0.18 C: (− 0.35, − 0.52 to 1.21) versus A/B (− 0.76, − 1.59 to 0.06), ns.; *p* = 0.07 ANOVA: n. s., *p* = 0.13 2. Ns. results for GF in treatment effect or change over time in arms, ns. difference in mean difference A/B versus C (0.57 − 0.49 to 1.64; *p* = 0.29); ns. mean differences for MFI, HADS and FACT-B between A/B and C
Smith et al. (2013)	RCT	Randomized (A/B/C): n = 30 (10/10/10) Drop-outs (A/B/C): 1 (1/0/0) Assessed (A/B/C): n = 29 (9/10/10) Breast cancer diagnosis	A: MA B: SA (Non-invasive sham needle (blunt- > no skin penetration) with the Park device at sham points, located on the lower back, abdomen, foot, lower leg and forearm) C. Waiting list control group 45 min. twice a week for 3 weeks and then once weekly for the last 3 weeks	T0: Baseline T1: After 2 weeks T2: After 4 weeks T3: After 6 weeks 1. Fatigue (BFI)	Mean BFI (SD): T0: A: 6.3 (1.7) B: 6.4 (1.3) C: 6.5 (0.9) T1: A: 3.9 (2.4) B: 6.1 (1.8) C: 6.0 (1.9) Mean difference (95% CI): 5.3 (4.5 to 6.2); *p* = 0.05 T2: A: 3.1 (2.7) B: 5.0 (1.8) C: 5.7 (2.5); Mean difference (95% CI): 4.6 (3.6 to 5.6); *p* = 0.06 T3: A: 3.2 (2.4) B: 4.9 (2.0) C: 5.4 (1.9) Mean difference (95% CI): 4.6 (3.6 to 5.5); *p* = 0.08
Molassiotis et al. (2007)	RCT	Randomized (A/B/C): n = 47 (15/16/16) Drop-outs (A/B/C): 11 (2/6/3) Assessed (A/B/C): n = 35 (13/9/13) Mixed cancer diagnoses; mostly lymphoma and breast cancer	A: MA B: Self-Acupressure (after training) C. Sham-Acupressure (Points LI12, GB33 and BL61, that are not associated with “energy” in the same way in traditional Chinese medicine) A: Six 20-min sessions over 2 weeks B/C: Daily for 2 weeks for 1 min at a time	T0: Baseline T1: After 2 weeks T2: After 4 weeks 1. Multidimensional Fatigue Inventory (MFI)	Mean (SD): T0: A: 16.4 (2.4); B: 16.6 (2.7); C: 7.8 (2.5) T1: A: 10.5 (3.0); B: 13.4 (3.0); C: 17.7 (2.6) T2: A: 12.8 (3.2); B: 14 (2.4); C: 16.9 (3.0) Improvement in A and B (ANCOVA shows group differences) *p* < 0.001; there was a 36% improvement in fatigue levels in A, while B improved by 19% and C only by 0.6%
Lu et al. (2012)	RCT	Randomized (A/B): n = 21 (11/10) Drop-outs (A/B): 6 (3/3) Assessed (A/B): n = 15 (8/7) Diagnosis of primary ovarian cancer; primary peritoneal cancer; papillary serous uterine cancer; or mixed mesodermal tumors of the uterus, ovary or fallopian tube	A: MA+electrostimulation B: SA (5 non-acupuncture points (9 needling sites) were located near verum points but outside the meridians. Once the needles were minimally inserted, no hand manipulation and no De Qi were allowed) +deactivated electrostimulation (an identical but deactivated EA stimulator was used) 10 sessions of acupuncture treatment, 2–3 times per week, for 30 min	T0: Baseline T1: After the acupuncture sessions (approx. 4 weeks) 1. Cancer-Quality-of-Life Questionnaire-Core 30 Item (EORTC-QLQ-C30) 2. Quality of Life Questionnaire–Ovarian Cancer Module-28 Item (QLQ-OV28)	After adjustment for baseline values, only sign. differences for scale SF in favor of A (*p* = 0.03); ns. differences for Global QoL mean (SD) at T1: A(n = 7): 69.0 (22.4); B(n = 7): 63.1 (17.3); *p* = 0.94 or fatigue at T1: A(n = 7): 34.9 (18.6); B(n = 7): 36.5 (8.4); *p* = 0.89
Deng et al. (2018)	RCT	Randomized (A/B): n = 63 (31/32) Drop-outs: 3 (2/1) Assessed (A/B): n = 60 (29/31) Multiple myeloma diagnosis	A: MA+Ctx B: SA (no needle inserted into the skin The acupuncturist attached an empty plastic acupuncture needle guide tube to the bone area next to each acupuncture point to create a perceptible sensation, and then stuck a needle to the skin surface with a piece of tape for 20 min)+Ctx 20 min. daily for 5 days	T0: Baseline T1: Day 1–5 T2: Day 15 T3: Day 30 1. MDASI	MDASI symptom score, mean (SD): Days 0 to + 5 (T0, T1): A: 1.23 (1.24); B: 1.66 (1.14); Difference:− 0.19; 95% CI:− 0.60 to 0.23; *p* = 0.4 T2: A: 1.34 (1.49); B: 2.10 (1.52); Difference:− 0.47; 95% CI:− 1.03 to 0.09; *p* = 0.1 T3: A: 1.15 (1.10); B: 1.67 (1.40); Difference:− 0.37; 95% CI:− 0.92 to 0.18; *p* = 0.2 A versus B: non-sign. for total MDASI core symptom scores and symptom interference scores during transplantation (*p* = 0.4 and 0.3, respectively) Sign. more effective in reducing nausea, loss of appetite and drowsiness: *p* = 0.042, 0.025 and 0.010 respectively
Hou et al. (2017)	RCT	Randomized (A/B/C): n = 169 (59/52/58) Drop-outs (A/B/C): 7 (2/3/2) Assessed (A/B/C): n = 162 (57/49/57) NSCLC diagnosis	A: TEAS B: Sham TEAS (treatment was applied to nearby areas outside the acupuncture points) C: Control group (no acupuncture) A/B: Sessions of 30 min. on days 1, 2, 3, 5, 8, 11, 14, 28	T0: Baseline T1: Day 8 T2: Day 28 1. RPFS	Mean (SD) T1: A: 2.85 ± 1.62; B: 2.59 ± 1.18; C: 2.68 ± 1.36; *p* = 0.615 T2: Sign. difference between the 3 groups: A: 2.06 ± 0.90; B: 2.80 ± 1.34; C: 3.00 ± 1.29; *p* < 0.01 All dimensions showed statistically significant differences, except for sensory fatigue (*p* = 0.50). However, A (mean = 2.48) had a lower score for sensory fatigue than C or B (mean = 3.20 and mean = 2.72)
					Multiple comparison functioning: A versus B: Behavioral: *p* = 0.919; Affective: *p* = 0.927; Sensory: *p* = 0.122; Cognitive: *p* = 0.795 A versus C: Behavioral: *p* = 0.001; Affective: *p* = 0.002; Sensory: *p* = 0.016; Cognitive: *p* < 0.01 B versus C: Behavioral: *p* = 0.031; Affective: *p* = 0.001; Sensory: *p* = 0.428; Cognitive: *p* = 0.016
Mao et al. (2014)	RCT	Randomized (A/BC): n = 67 (21/20/26) Drop-outs (A/B/C): 8 (1/2/1 (8w), 2/1/1 (12w)) Assessed (A/B/C): n = 59 (18/17/24) History of early breast cancer (stage I-III)	A: Electro-acupuncture B: Sham Electro-acupuncture (Non-penetrating Streitberger needles at non-acupuncture and non-trigger points, at least 5 cm from the joint where pain was felt to be maximal. The acupuncturists avoided eliciting the “de qi” sensations by minimally manipulating the needles apart from their initial contact with the skin TENS unit on a different channel so that the subject could watch the light blink without receiving any current) C: Control group with normal care on the waiting list twice a week for 2 weeks, then weekly for a further 6 weeks	T0: Baseline T1: Week 2 T2: Week 4 T3: Week 8 T4: Week 12 1. BFI	Sign. improvement in A compared to C across all time points (mixed model; *p* = 0.0095), with sign. differences at T3 and T4; Change from T0 (mean, 95% CI): T2: A: − 0.4 (− 1.6 to 0.7); B: − 0.5 (− 1.7 to 0.7); C: − 0.1 (− 0.8 to 0.6); A versus C: − 0.3 (− 1.6 to 1.0, *p* = 0.57); B versus C: − 0.4 (− 1.8 to 1.0, *p* = 0.38) T3: A: − 1.4 (− 2.7 to − 0.1); B: − 0.6 (− 1.7 to 0.5); C: 0.5 (− 0.2 to 1.3); A versus C: − 2.0 (− 3.4 to − 0.5, *p* = 0.0034); Cohen d = 0.96; B versus C: − 1.2 (− 2.5 to 0.1, *p* = 0.046) T4: A: − 1.4 (− 2.7 to − 0.1); B: − 0.7 (− 1.6 to 0.1); C: 0.2 (− 0.8 to 1.2); A versus C: − 1.6 (− 3.2 to − 0.07, *p* = 0.022); Cohen d = 0.86; B versus C: − 0.9 (− 2.2 to 0.3, *p* = 0.091)

### Excluded studies

Excluded were 14 RCTs because they did not meet the inclusion criteria. Most of them didn’t meet the criteria for being a randomized controlled trial, didn’t investigate fatigue or used acupuncture as stand-alone treatment. A list of excluded studies can be seen in Table 4.

**Table 4:** Table of excluded studies

References	Study type	Year	Title	Reason for exclusion
Zhang et al. (2023)	RCT	2023	Acupuncture for chemotherapy-associated insomnia in breast cancer patients: an assessor-participant blinded, randomized, sham-controlled trial	No explicit values for fatigue listed
Kim et al. (2015)	RCT	2015	Acupuncture for chronic fatigue syndrome and idiopathic chronic fatigue: A multicenter, nonblinded, randomized controlled trial	Wrong sample (Not only cancer patients)
Li et al. (2023b)	RCT	2023	Governor vessel moxibustion for cancer-related fatigue in colorectal patients: a randomized trial	Wrong topic (Moxibustion)
Stie et al. (2022)	RCT	2022	Impact of Open Dialogue about Complementary Alternative Medicine—A Phase II Randomized Controlled Trial	Wrong topic (No acupuncture treatment)
Zick et al. (2016)	RCT	2016	Investigation of 2 Types of Self-administered Acupressure for Persistent Cancer-Related Fatigue in Breast Cancer Survivors: A Randomized Clinical Trial	Wrong topic (Acupressure)
Lv et al. (2022)	RCT	2022	Acupuncture ameliorates breast cancer-related fatigue by regulating the gut microbiota-gut-brain axis	Wrong sample (mice)
Wang et al. (2022)	MA	2022	Acupuncture and acupressure with improved cancer-related depression of retrospective studies	Wrong study type (not an RCT)
Halamkova et al. (2022)	Report	2022	Acupuncture from the perspective of evidence-based medicine—options of clinical use based on National Comprehensive Cancer Network (NCCN) guidelines	Wrong study type (not an RCT)
Witt (2014)	Abstracts	2014	Acupuncture in oncology-the current evidence	Wrong study type (not an RCT)
Harris et al. (2017)	RCT	2017	Brain Connectivity Patterns Dissociate Action of Specific Acupressure Treatments in Fatigued Breast Cancer Survivors	Wrong topic (Acupressure)
Berger (2013)	Article	2013	Does the strength of evidence support recommending acupuncture to relieve cancer-related fatigue?	Wrong study type (not an RCT)
Salmon (2013)	Descriptive study	2013	Evaluation of an acupuncture service in oncology	Wrong study type (not an RCT)
Zhang et al. (2021)	SR	2021	The positive role of traditional Chinese medicine as an adjunctive therapy for cancer	Wrong study type (not an RCT)
Hoxtermann et al. (2021)	RCT	2021	Efficacy and Safety of Auricular Acupuncture for the Treatment of Insomnia in Breast Cancer Survivors: A Randomized Controlled Trial	Wrong topic (Auricular Acupuncture)

### Risk of Bias and GRADE ratings of the included studies

**Fig. 2:**
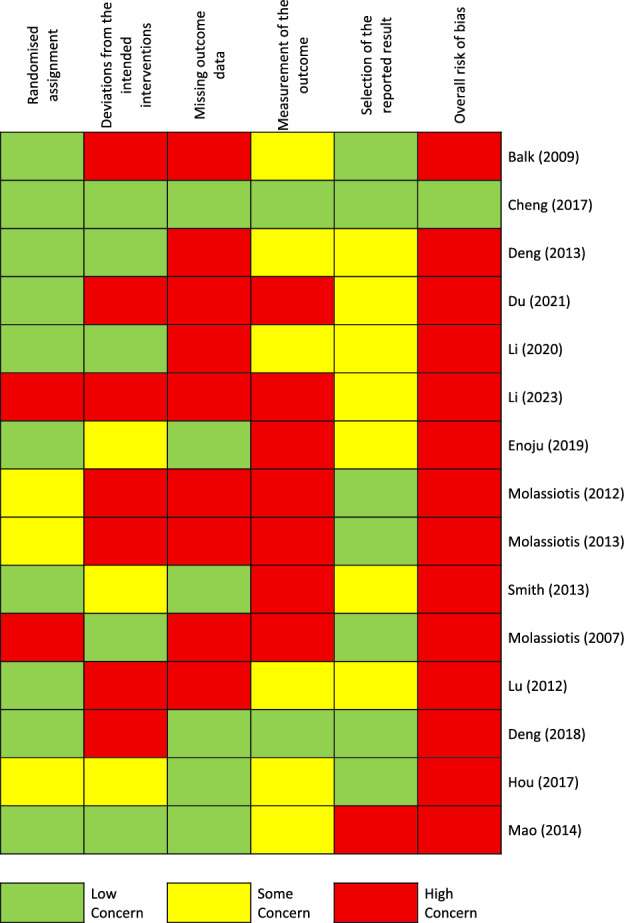
Risk of bias assessment (RoB tool 2.0.)

In the RoB assessment using the RoB-2 tool Sterne, Savović (Sterne et al. 2019), 14 of 15 studies were classified as “high concerns’”, one as “low concerns” and none as “some concerns” (Fig. 2). Common methodological problems were insufficient information on blinding (e.g. insufficient information on allocation sequence concealment), which was particularly important, as questionnaires also require patients to act as assessors Table 5. In addition, appropriate analysis, like Intention-to-treat (ITT), was often not used to estimate the effect of allocation or insufficient information on data analysis was provided.

**Table 5:** Table risk of bias

Reference	Study type	Standardized rating of risk of bias	Additional comments on methodology
Balk et al. (2009)	RCT	Domain 1: low Domain 2: high Domain 3: high Domain 4: some Domain 5: low Overall: high	PRO: Methodical quality: Institutional Review Board approved; all subjects suspected that they had received genuine acupuncture at the 3rd and 10th week follow-ups; Inclusion of confounders in analysis
			CONTRA: Sample: Small sample (n = 22) Methodical quality: No ITT; one of the two acupuncturists was the author of the article; no mean values/medians/p-values at individual time points given except T0; possibly not real acupuncture as the needles were also fitted with tubes
Cheng et al. (2017)	RCT	Domain 1: low Domain 2: low Domain 3: low Domain 4: low Domain 5: low Overall: low	PRO: Sample: Small size statistically considered Methodical quality: Credibility questionnaires for blinding verification; Intention-to-Treat Population (ITT); Ethics Council approved; Acupuncture STRICTA protocolled
			CONTRA: Sample: Small sample (28) Methodical quality: All interventions carried out by the same acupuncturist; possible influence through financing
Deng et al. (2013)	RCT	Domain 1: low Domain 2: low Domain 3: high Domain 4: some Domain 5: some Overall: high	PRO: Sample: Adequate size (n = 101), adjusted for baseline characteristics Methodical quality: Randomization stratified for baseline BFI > 6; blinding was checked; ITT analysis, power analysis was conducted
			CONTRA: Sample: High dropout rate (A/B n = 15/11) Methodical quality: Possible influence by acupuncturists Report quality: No detailed information about test results
Du et al. (2021)	RCT	Domain 1: low Domain 2: high Domain 3: high Domain 4: high Domain 5: some Overall: high	PRO: Methodical quality: Ethics council approved; Shariro-Wilk test
			CONTRA: Sample: Dropouts unclear Methodical quality: No blinding; no Sham acupuncture (placebo effect can’t be ruled out), no power- or ITT analysis, Report quality: No exact data at what time the baseline data were collected; no comparability in the emotional field
Li et al. (2020)	RCT	Domain 1: low Domain 2: low Domain 3: high Domain 4: some Domain 5: some Overall: high	PRO: Sample: Group size > 30 (pilot study) Methodical quality: Per-protocol basis; ITT analysis (primary endpoints); ethics council approved; testing of blinding
			CONTRA: Methodical quality: Funded Report quality: Results difficult to understand due to missing values at the time points and little information on statistical differences
Li et al. (2023a)	RCT	Domain 1: high Domain 2: high Domain 3: high Domain 4: high Domain 5: some Overall: high	PRO: Sample: sample size > 30 Methodical quality: Ethic committee; authors declare no conflict of interest
			CONTRA: Sample: not big enough; monocentric Methodical quality: short investigation time; no ITT analysis; funded; open— > no sham acupuncture
Eonju et al. (2019)	RCT	Domain 1: low Domain 2: some Domain 3: low Domain 4: high Domain 5: some Overall: high	PRO: Methodical quality: Ethics Council approved; one acupuncturist
			CONTRA: Sample: Small sample size Methodical quality: No comparability because acupuncture points were determined individually; no ITT analysis; only woman; short follow-up time
Molassiotis et al. (2012)	RCT	Domain 1: some Domain 2: high Domain 3: high Domain 4: high Domain 5: low Overall: high	PRO: Sample: big(n = 302) Methodical quality: ITT analysis; ethic committee approved; results checked with therapists’ reports
			CONTRA: Sample: Not all assessed completed all of the treatments Methodical quality: Not blinded (placebo bias); no baseline data
Molassiotis et al. (2013)	RCT	Domain 1: some Domain 2: high Domain 3: high Domain 4: high Domain 5: low Overall: high	PRO: Sample: 151 Methodical quality: Ethic approved; power analysis; STRICTA
			CONTRA: Sample: High drop-out (23, 35%) Methodical quality: No ITT analysis; follow-up study—may have been influenced; missing outcome values
Smith et al. (2013)	RCT	Domain 1: low Domain 2: some Domain 3: low Domain 4: high Domain 5: some Overall: high	PRO: Methodical quality: Analysis examined the demographic and baseline characteristics; ethic committee approved; ITT analysis; STRICTA Checklist; testing of blinding
			CONTRA: Sample: Small sample size (< 30) because pilot study, 2 assessed participants didn’t complete all 9 acupuncture treatments Methodical quality: Differing acupuncture points; missing outcome values; short observation time; only blind for 2 of 3 groups
Molassiotis et al. (2007)	RCT	Domain 1: high Domain 2: low Domain 3: high Domain 4: high Domain 5: low Overall: high	PRO: Sample: Sample size > 30 Methodical quality: Ethic committee approved; ITT principle; STRICTA; one acupuncturist for all
			CONTRA: Sample: Too small according to the power analysis Methodical quality: Funded; no direct group comparison
Smith et al. (2013)	RCT	Domain 1: low Domain 2: high Domain 3: high Domain 4: some Domain 5: some Overall: high	PRO: Methodical quality: Blinding assurance through validated credibility scale; same contact time contact time between the treating acupuncturists and the patients; visual barrier to block patient's view of the needle sites
			CONTRA: Sample: Small sample size (14); Drop-out 29% (unclear due to different numbers of results) Methodical quality: No ITT analysis; short observation time
Deng et al. (2018)	RCT	Domain 1: low Domain 2: high Domain 3: low Domain 4: low Domain 5: low Overall: high	PRO: Methodical quality: Patients’ eyes covered with plasters for better blinding; ethic committee approved; blinding tested with high credibility values; power analysis
			CONTRA: Methodical quality: No ITT analysis; hardly any baseline information; no values for scales outside the significant range
Hou et al. (2017)	RCT	Domain 1: some Domain 2: some Domain 3: low Domain 4: some Domain 5: low Overall: high	PRO: Methodical quality: Groups comparable at baseline; ethic committee approved; power analysis
			CONTRA: Methodical quality: Participants’ daily activities may have affected their fatigue levels; no ITT analysis; no precise information on blinding or side effects Report quality: Collecting data only three times within 28 days could increase retention, which could affect the results
Mao et al. (2014)	RCT	Domain 1: low Domain 2: low Domain 3: low Domain 4: some Domain 5: high Overall: high	PRO: Methodical quality: ITT between the EA and WLC arms and between the SA and WLC arms based on interactions between time and intervention in the mixed-effects models; Groups comparable to baseline; approved by Institutional Review Board; proof of blinding (Credibility assessment in week 8); power analysis
			CONTRA: Methodical quality: A versus B not reported; Two licensed; non-medical acupuncturists with 8 and 20 years of experience -> very different

The overall certainty of the evidence for the outcome fatigue was additionally assessed using the GRADE approach (Table 6). Consistent with the RoB-2 results, the overall certainty of evidence in the studies was very low, mainly due to serious concerns about risk of bias, imprecision, and, in some cases, indirectness of outcome measures.

**Table 6:** Grade

Certainty assessment							Effect	Certainty	Importance
No. of studys	Design	Risik of bias	Inconsistency	Indirection	Precision	Other factors			
Fatigue (assessed with various fatigue scales (BFI/BFI-C/PFS/RPFS/VAS-F/MFSI/FSS/MFI/MFI-20/FACIT-F/EORTC-QLQ-C30/MDASI))									
15	Randomized clinical Trials	Very serious^a^	Very serious^b^	Serious^c^	Serious^d^	Serious suspection for publication bias	RCTs report mixed effects; ten individual trials show small to significant reductions in fatigue, five studies found no advantage for acupuncture. The high heterogeneity and low RoB ratings limit interpretability	⨁◯◯◯ Very low^a,b,c,d,e^	Critical

^a^14/15 studies show high RoB (RoB-2); only 1 low

^b^Heterogeneous results across studies, partially conflicting

^c^Different populations (during/post therapy), various scales

^d^Small sample sizes, low power, few studies with ITT analysis

^e^Some studies were funded by institutions with a potential interest in positive results, increasing the likelihood of publication bias

Overall, the high risk of bias, low GRADE score, and significant clinical and methodological heterogeneity are strong arguments against the possibility of performing a meta-analysis. Pooling data from such heterogeneous and low-certainty studies would not lead to meaningful or reliable conclusions about the efficacy of acupuncture for cancer fatigue.

### Efficacy of acupuncture therapy

Fatigue was assessed with the Brief Fatigue Inventory (BFI) (Deng et al. 2013; Smith et al. 2013; Mao et al. 2014), the Chinese version of the Brief Fatigue Inventory (BFI-C) (Cheng et al. 2017), the Piper Fatigue Scale (PFS) (Du et al. 2021), the Revised Piper Fatigue Scale (RPFS) (Hou et al. 2017), the Visual Analogue Scale to Evaluate Fatigue Severity (VAS-F) (Li et al. 2020), the 20item Multidimensional Fatigue Symptom Inventory (MFSI) (Li et al. 2023a), the Fatigue Scale Score (FSS) (Eonju et al. 2019), the Multidimensional Fatigue Inventory (MFI) (Molassiotis et al. 2012, 2007), MFI-20 (Molassiotis et al. 2013) and the Functional Assessment of Chronic Illness Therapy-Fatigue (FACIT-F) (Balk et al. 2009). Two studies looked at fatigue only in a broader context as part of the European Organisation for Research and Treatment of Cancer Quality of Life Questionnaire Core 30 (EORTC-QLQ-C30) (Lu et al. 2012) and within the MD Anderson Symptom Inventory (MDASI) (Deng et al. 2018).

In the only study with a low risk of bias by Cheng et al. (2017) significant fatigue reduction was observed after 6 weeks in the acupuncture group compared to the sham acupuncture group (*p* < 0.001) in lung cancer patients. Further nine studies, all rated with high risk of bias (Du et al. 2021; Li et al. 2020; Li et al. 2023a; Eonju et al. 2019; Molassiotis et al. 2012, 2007; Smith et al. 2013; Hou et al. 2017; Mao et al. 2014), also conclude a significant positive effect from acupuncture on cancer treatment related fatigue. Du et al. (2021) found that acupuncture significantly reduced fatigue scores after 3 weeks, particularly improving perception dimension (mental state), in chemotherapy-induced fatigue patients (n = 26) after intestinal cancer compared to controls (n = 24). Li et al. (2020) reported that ATAS acupuncture improved fatigue, anxiety, and insomnia over 3–4 paclitaxel cycles (n = 20) compared to the sham (n = 10) and control (n = 10) groups (*p* = 0.004). In another study by the same author (Li et al. 2023a), including breast cancer survivors over 3–4 radio- or chemotherapy cycles, lower fatigue values and a higher performance status was found for patients in the mind-regulation acupuncture group compared to control (both groups received ShenqiFuzheng injections). Two other studies investigated breast cancer patients after therapy and also found an advantage for the acupuncture group after 2 (Smith et al. 2013) and 6 weeks (Molassiotis et al. 2012) compared to the waitlist or control group (*p* < 0.001; *p* = 0.05). A study by Eonju et al. (2019) found an advantage of acupuncture in improving fatigue scores in patients after thyroidectomy over 6 weeks compared to control group. One study compared acupuncture to an acupressure and a sham acupressure group and found significant improvements at the end of intervention (2 weeks) with regards to general fatigue (*p* < 0.001) and physical fatigue (*p* = 0.016) for the first two groups, in patients with a history of chemotherapy for different types of cancer (Molassiotis et al. 2007). Improvements were observed even 2 weeks after treatments, although they were lower. The last two studies (Hou et al. 2017; Mao et al. 2014) were using electroacupuncture. Hou et al. (2017) found significant improvements from Transcutaneous Electrical Acupoint Stimulation (TEAS) acupuncture (n = 57) after 28 days compared to the control (n = 56) and sham groups (n = 49) for affective fatigue (*p* < 0.01), sensory fatigue (*p* = 0.05) and cognitive fatigue (*p* < 0.01) in non-small-cell lung cancer (NSCLC) patients under chemotherapy. In Mao et al. (2014) the electro-acupuncture group (n = 22) showed significant improvements compared to the sham group (n = 22), including woman with a history of breast cancer under aromatase inhibitor therapy over 2, 4 and 8 weeks.

Five Studies (Deng et al. 2013, 2018; Molassiotis et al. 2013; Lu et al. 2012; Balk et al. 2009) found no advantage for acupuncture. Two studies (Deng et al. 2013; Balk et al. 2009) reported no significant differences between acupuncture (n = 16; 49) and sham group (n = 11; 52) over 6 (Balk et al. 2009) or 3, 6 or 10 weeks (Deng et al. 2013). Deng et al. (2018) found nonsignificant reductions in symptom scores and symptom interference scores for true acupuncture patients compared to sham patients under chemotherapy for multiple myeloma over approximately 8 weeks. Molassiotis et al. (2013) found no significant results of the further acupuncture sessions at 18 weeks beyond the improvement observed in initial trial (Molassiotis et al. 2007) between acupuncture (n = 49), self-acupuncture (n = 56) and patients without further acupuncture (n = 46). Lu et al. (2012) found improved subscores in the acupuncture arm (n = 8) after about 4 weeks. However, after adjusting for baseline differences, only the SF score was significantly higher compared with the sham acupuncture arm (n = 7) (*p* = 0.03) and there were no significant results concerning fatigue (*p* = 0.89).

### Adverse events

Only few side effects like light pain/discomfort (Cheng et al. 2017; Molassiotis et al. 2013; Molassiotis et al. 2007; Mao et al. 2014), dizziness (Cheng et al. 2017), (spot) bleeding (Cheng et al. 2017; Molassiotis et al. 2013, 2007), slight bruising (Li et al. 2020; Molassiotis et al. 2007; Mao et al. 2014), nausea and nervousness (Molassiotis et al. 2007) were reported. Many studies reported no adverse events at all (Deng et al. 2013, 2018; Du et al. 2021; Eonju et al. 2019; Hou et al. 2017; Lu et al. 2012; Balk et al. 2009) and some gave no information on side effects (Li et al. 2023a; Molassiotis et al. 2012; Smith et al. 2013).

### Risk of bias assessments in acupuncture studies with sham control

Most of the included studies are high risk when assessed using the Cochrane RoB tool 2.0. and only 1 study has low concerns. And even for this one the RoB 2 tool may not take into account all aspects that need to be explicitly considered when assessing the reliability of a study on acupuncture. The following aspects may lead to unblinding or patients knowing which arm they are in:

- Different number of points in RA and SA: Smith et al. (2013)
- Different penetration depth in RA and SA: Cheng et al. (2017), Li et al. (2020)
- Non-penetrating needles—sometimes fixed with adhesive tape (as SA): Balk et al. (2009), Deng et al. (2013), Smith et al. (2013), Deng et al. (2018), Mao et al. (2014)
- Different localization of the points: Deng et al. (2013), Li et al. (2020), Smith et al. (2013), Molassiotis et al. (2007) (acupressure), Lu et al. (2012) (visual barrier), Deng et al. (2018) (eyes covered), Mao et al. (2014)
- In case of EA: current applied in RA versus no current in SA: Balk et al. (2009), Lu et al. (2012), Hou et al. (2017), Mao et al. (2014)
- The deqi-feeling triggered in RA not in SA: Balk et al. (2009), Cheng et al. (2017), Deng et al. (2013), Smith et al. (2013), Lu et al. (2012), Mao et al. (2014)

Moreover, acupuncture can come with intense interaction between patient and acupuncturist. Therefore, differences in procedure with less interaction in one arm might not lead to unblinding but to a weaker response, which would explain differences between verum and sham groups independent of a specific effect of acupuncture. The positive effect in the RA arm can be described as an attention and interaction effect, which is more than a placebo effect:

- The deqi-feeling triggered in the RA group to find the exact puncture point: Cheng et al. (2017), Deng et al. (2013), Smith et al. (2013), Lu et al. (2012), Mao et al. (2014), Eonju et al. (2019)
- Open or hidden differences in setting and attention/attitude of the acupuncturist: Balk et al. (2009), Cheng et al. (2017), Smith et al. (2013), Lu et al. (2012), Hou et al. (2017), Mao et al. (2014)
- Individual points for patients or semi-standardized protocols: Smith et al. (2013)

Some authors try to detect unblinding by asking participants after the intervention whether they think they were in the RA or SA group (Balk et al. 2009; Cheng et al. 2017; Deng et al. 2013; Li et al. 2020; Deng et al. 2018) or using a credibility scale (Lu et al. 2012) or rating (Mao et al. 2014). However, this question does not reveal the influence of a more intense interaction, as the patient may believe to be in the verum arm in both cases. In Mao et al. (2014), patients in a wait list control group were able to receive 10 RA sessions after their follow-up, there is no information on how many patients took advantage of this. Control groups with a waiting list may also be subject to bias, as patients may expect their symptoms to improve only after this waiting period.

## Discussion

In our systematic review, we found significant limitations in the methodological quality of the available studies on acupuncture for cancer therapy-induced fatigue. Several methodological challenges were common and often led to a high risk of bias, limiting the reliability of the results. In addition, the quality assessment criteria we used further reduced the reliability of these studies, which increased concerns about the strength of the evidence.

Fatigue, particularly in the context of cancer treatment, is inherently subjective, with everyone’s experience being shaped by various physical, emotional and psychosocial factors. This subjective nature presents challenges when evaluating treatments such as acupuncture, where the perceived benefits can be influenced by the therapeutic attention and care that patients receive during sessions. The attention and interaction inherent in acupuncture treatments may themselves have a positive impact on fatigue symptoms, as this personalized care may promote a sense of relief and support that patients associate with the effectiveness of the treatment. The interaction between patient and practitioner could particularly confound the results of the studies with only a control group and no sham group, making it difficult to distinguish between the physiological effects of acupuncture and those arising from the care context.

To rigorously evaluate the efficacy of acupuncture for cancer-related fatigue, studies with three-arm designs-comparing real acupuncture (RA), sham acupuncture (SA), and a non-intervention control group are therefore essential. Of the studies included in our review, only one met these design criteria (Hou et al. 2017), which reported positive effects of acupuncture on fatigue. However, their results should be interpreted with caution due to a high risk of bias rating and limited robustness of the study design. But even with ideal study design and blinding, sham interventions may not be physiologically inert, as any form of somatosensory stimulation, even outside traditional acupuncture points, could produce therapeutic effects. This challenges the strict separation between real and sham acupuncture and may lead to an underestimation of verum acupuncture’s true efficacy. This view also raises fundamental questions about the core principles of acupuncture: If any peripheral stimulation can trigger therapeutic responses, it becomes unclear what specifically constitutes acupuncture. Acupuncture points are not uniformly defined, and there is no clear anatomical or biophysical evidence for the concept of meridians or qi. These considerations emphasize the need to critically re-evaluate the theoretical assumptions of acupuncture and to further investigate which components of the intervention could be responsible for the observed therapeutic effects.

At the same time, ensuring effective blinding remains one of the main methological challenges in acupuncture research. Patients, especially those familiar with acupuncture, may perceive subtle cues that reveal their assigned treatment. Differences in the depth of needling, the number of needles used, or the precise points targeted in RA versus SA may be recognizable to observant participants. In addition, the RA group often follows semi-standardized or individualized protocols, while SA typically follows strict, standardized protocols, which may give patients additional clues as to which treatment they are receiving. Similarly, non-penetrating sham acupuncture needles, which are commonly stuck to the skin, may introduce cues that could influence patient perception and response. Even if patients are unaware of these specific differences, the different interactions and context of treatment in RA versus SA may influence outcomes through placebo or expectation effects. The challenge for efficient blinding further arises from the interaction between patient and acupuncturist, because it is not possible to blind the acupuncturist, who must distinguish between real and sham acupuncture points (RA and SA). This awareness may inadvertently influence patient interactions and potentially enhance therapeutic effects if the acupuncturist is more convinced of the efficacy of RA compared to SA. The use of electroacupuncture brings additional challenges in maintaining blinding. For example, Hou et al. (2017) adjusted the electrical intensity until patients felt a slight muscle twitch, a sensation that is impossible to replicate in sham treatments. Such differences inherently jeopardize blinding, as sensations such as these are unique to true electroacupuncture. In traditional acupuncture the “de qi” sensation—a unique tingling or heaviness felt by the patient when the needles are inserted-is frequently elicited and is an integral part of point identification. Because the patient must acknowledge the occurrence of this sensation, this interactive component not only personalizes the treatment, but also encourages a more engaged and potentially therapeutic interaction between patient and acupuncturist. This reliance on patient feedback again raises questions about the true source of the therapeutic effects: whether they stem from precise needle placement at acupuncture points or from the complex dynamic between patient and practitioner, possibly influenced by the patient’s perception of personalized care and active involvement. This “de qi” feeling was actively sought in the RA groups in six of the studies and could potentially bias the results in favor of RA over SA. This is because this focus on achieving de qi could contribute to RA appearing more effective than SA, as observed in four studies (Wissenschaftlichen et al. 2024; Li et al. 2023a; Molassiotis et al. 2013; Hou et al. 2017). And even among these studies, not all observed an association, as two studies found no significant differences between RA, SA and control groups (Cheng et al. 2017; Mao et al. 2014). In the SA groups, efforts were made to prevent the de qi sensation, either by minimizing needle stimulation (Hou et al. 2017) or by avoiding additional manipulations altogether, but these modifications alone cannot completely prevent the influence of patient expectations or placebo effects on outcomes, as reflected by the fact that SA groups often showed a difference compared to control groups.

Sample sizes were small in many studies, which reduces statistical power and limits generalizability. In addition, many studies reported high dropout rates and missing outcome data, which could potentially affect the validity of their conclusions. Also, some studies used a per-protocol analysis approach instead of an intention-to-treat analysis, which limits the reliability of the results by potentially overestimating treatment effects as non-compliant or withdrawn participants were excluded. Because of these design flaws and inadequate controls, from the fifteen included studies, only one was rated at low risk of bias in our review (Cheng et al. 2017), using the RoB2 tool (Eldridge, et al. 2016). Still, this study showed additional limitations not covered by the RoB Tool. Importantly, it lacked power analysis and included only 28 participants, which reduced its statistical power and generalizability. Furthermore, this study received funding from the Comprehensive and Integrative Medicine Institute (CIMI) and the National Natural Science Foundation of China, which introduces a potential conflict of interest. If these additional limitations were included in our risk assessment tool, all RCTs in this review would likely be categorized as high risk, highlighting the need to refine the RoB criteria to more effectively identify such critical factors.

To address methodological weaknesses in acupuncture research, the STRICTA (Standards for Reporting Interventions in Clinical Trials of Acupuncture) checklist was introduced in 2001 and updated in 2010 (MacPherson et al. 2010). STRICTA includes six essential criteria for detailing acupuncture interventions, with the aim of improving transparency and reproducibility in acupuncture research. Although this reporting standard was available during the timeframe of the studies reviewed, only five of the fifteen included RCTs referenced STRICTA in their methodology sections (Wissenschaftlichen et al. 2024; Eonju et al. 2019; Molassiotis et al. 2012, 2013; Smith et al. 2013). Notably, despite following STRICTA guidelines, four of these five studies still received a ‘highly questionable’ rating in our risk assessment (RoB), indicating that adherence to STRICTA alone may not significantly improve the methodological quality of acupuncture studies. Our results suggest that although STRICTA is beneficial for standardizing the reporting of intervention details such as acupuncture points used and the generation of de qi sensation, it does not provide comprehensive guidelines for reducing bias. For example, STRICTA does not include specific recommendations for minimizing performance or detection bias, which are critical in acupuncture studies where blinding is difficult. In all five studies that adhered to STRICTA, de qi sensation was elicited in the RA group, with no corresponding procedures used in the SA groups. This discrepancy could bias the results a priori, as the de qi sensation could influence patients’ perceptions and therapeutic expectations, potentially skewing the results in favor of RA.

To increase the reliability and comparability of future acupuncture studies, it would be useful to adapt the RoB instrument for acupuncture-specific requirements. A supplementary checklist with the criteria developed in this study could contribute to a more precise identification of biases. Recommended factors include specific measures for blinding patients and data analysts, consistency in the application of the de-qi sensation across study arms, and precise control of practitioner-patient interactions. Such a specialized approach would not only improve the quality of systematic reviews but also promote the comparability of studies investigating the same therapeutic outcomes.

In addition, the subjective nature of fatigue should be considered as it may cause unintended effects when acupuncture is used in place of evidence-based interventions such as exercise, which has been shown to be effective in reducing fatigue in cancer patients. Regular physical activity is well documented and offers clear benefits in terms of physical performance, mental health and energy levels. However, when patients choose acupuncture as their main approach to fatigue management, they may tend to forgo proven and active approaches such as exercise (Wissenschaftlichen et al. 2024).

Moreover, mind–body interventions such as yoga, tai chi, and qigong have also shown promising effects on cancer-related fatigue. A Cochrane meta-analysis by Cramer et al. from 2017 found moderate evidence that yoga leads to a significant reduction in fatigue in cancer patients, especially breast cancer survivors, compared to no treatment or psychosocial/educational interventions (Cramer et al. 2017). Similarly, Wayne et al. (2018) reported significant improvements in fatigue, sleep quality, depression, and overall quality of life in cancer patients practicing tai chi or qigong, with moderate effect sizes observed for fatigue.

This emphasizes the need to critically evaluate not only the benefits of acupuncture, but also its impact on patients’ willingness to implement other evidence-based interventions. Future studies should therefore consider a study design that directly compares acupuncture with exercise as a fatigue management therapy. A three-arm design in which patients are randomized to receive acupuncture, participate in a structured exercise program or are assigned to a non-intervention control group could provide valuable insights. Assessment of physiological and subjective outcomes would show whether acupuncture offers specific benefits over exercise or whether the beneficial effect is mainly due to the attention and care components of acupuncture. In addition, comparison of adherence and satisfaction between these groups could indicate whether the acceptance of acupuncture is due more to the holistic care aspects than to specific therapeutic effects.

### Limitations of this work

Limitations of this work are that only articles in German and English have been examined. It was also only possible to search databases in these two languages.

## Conclusion

We are unable to make firm judgments regarding the efficacy of acupuncture in treating cancer-related fatigue due to the inconsistent outcomes and methodological constraints of the current research. The total data is still insufficient despite the inclusion of 15 researches because of several issues with study design and contradictory findings. This analysis emphasizes that in order to fully comprehend the role of acupuncture in the treatment of chemotherapy-induced fatigue, future research must employ more exacting designs and thorough evaluation instruments.

## Supplementary Information

Below is the link to the electronic supplementary material.

Supplementary file1 (DOCX 96 KB)

## Data Availability

No datasets were generated or analysed during the current study.
