# Supplementary material for: Acupuncture as treatment of cancer-therapy induced fatigue: a critical systematic review with a focus on the methodological assessment of blinding
Source: J Cancer Res Clin Oncol. 2026 Jan 8;152(1):37. doi: 10.1007/s00432-025-06395-4 (PMC12783427; doi:10.1007/s00432-025-06395-4)
Supplement: Supplementary file 1 — Supplementary file1 (DOCX 96 KB) [file 432_2025_6395_MOESM1_ESM.docx]

| Referenz | Studientyp | Patienten-merkmale | Intervention/  Kontrolle, Beobachtung | Untersuchte Endpunkte | Hauptergebnisse | Neben-/ Wechselwir­kungen | Finanzierung/ Interessens-konflikte | Methodische Bemerkungen | Evidenz-klasse (Oxford) |
| --- | --- | --- | --- | --- | --- | --- | --- | --- | --- |
| Balk (2009):  Pilot, randomized, modified, double-blind, placebo-controlled trial of acupuncture for cancer-related fatigue. Journal oft he Society for Integrative Oncology  [1] | RCT  Prospektiv  Monozentrisch  Doppelt verblindet (Pat., staff,  assessors)  2 Arme  Eingeschlossen: N=27  Ausgewertet:  N=22  Drop-out: A/B n = 2/3 (1 wegen Arbeitsverantwortung, etc.)  Land : USA/Pittsburgh  Magee-Womens Hospital Department of Radiation Oncology  November 2005 bis Juni 2007 (Auswählen der Patienten) | Alter(MW, Range):  54.1, 40-69  Geschlecht: 100% weiblich  Frauen mit lokalisiertem Krebs (alle Brustkrebs, nur eine mit Endometrialkarzinom), die allein oder in Kombination mit einer Chemotherapie operiert wurden und eine Strahlentherapie planten (6 Wochen)  FACIT-F <= 44  Ohne  Akupunkturerfahru  ng in  Vergangenheit | **Arm A:**  N=16  Manuelle Körper-akupunktur  6-4 Wochen, 2 x wöchentlich für 30 min.  Akupunkturpunkte: Ki-3, Sp-6, LI-4, St-36, und Ren-6/CV-6. Ki-3  Der Akupunkteur versuchte, das Gefühl eines „Nadelgreifens“ zu erzeugen, jedoch ohne  Feedback von  Patient  niederfrequente Elektrostimulation (1 Hz) an Ki-3(-) und St-36(+)  Eine Wärmelampe wurde über dem CV-6-Punkt am Unterbauch platziert  „de qi“ Aufklärung  **Arm B:**  N=11 (Sham Akupunktur mit Park Sham Device, einer stumpfen Nadel, Elektrosimulation, Wärmelampe auf schwach)  Lizensierte  Akupunkteure  (Dr. Balk, Dr.  Kalro) | T0: Vor der ersten Bestrahlungssitzung und Akupunktursitzung  1.,2.,3. + soziodemografischer Fragebogen  T1: 3 Wochen (1.)  T2: 6 Wochen(Ende des Bestrahlungsregiments (1.,2.,3.)  T3: 10 Wochen (1.,2.,3.)  **Primäre Endpunkte:**  1. Krebs-assoziierte Fatigue mit FACIT-F  **Sekundäre Endpunkte:**  Lebensqualität und Krebs-assoziierte Fatigue Belastung mit  2. Form-36 (SF-36) und  3. dem Cancer Related Fatigue Distress Scale (CRFDS)  **Konfundierte-Messungen:**  4. Brief Symptom Inventory 18 (BSI 18)  5. Center for Epidemiological Studies Depression Scale (CES-D)  6. Physical Activity Questionnaire | ANOVA Analyse  Zu 1.: T0:  Mittelwert/Median  A: 31.9/31  B: 36.6/38  Difference (Follow-Up-Baseline)  Repeated Measures ANOVA:  Treatment (p=0.457) Time (p=0.106)  Zu 2.:  Repeated Measures ANOVA:  PCS-Score:  Treatment p=0.972  Time p=0.370  MCS-Score:  Treatment p=0.197 Time p=0.454  Veränderung  innerhalb Gruppe  oder  Gruppendifferenz  zu keinem  Zeitpunkt sign.,  Vergleich Different  zu T0 zwischen  den Gruppen über  alle Zeitpunkte,  Gruppeneffekt  p=0.457, Zeiteffekt  p=0.106, ns.  Zu 3.:  Repeated Measures ANOVA:  Mean CESD Scores:  Treatment p=0.294 Time p<0.009  Veränderung  innerhalb Gruppe  oder  Gruppendifferenz  zu keinem  Zeitpunkt sign.;  Gruppeneffekt  p=0.972, Zeiteffekt  p=0.370, ns.  Zu 4.: keine Mittelwerte/Mediane usw. angegeben, kein p-Wert angegeben  Zu 5.:  Mittelwert/Median  A: 21.6/21.5  B: 21.8/16 | Laut Autoren kam es bei keinem der Probanden zu Nebenwirkungen | Finanzierung: Diese Studie wurde vom National Cancer Institute finanziert. Veröffentlichung durch Mittel des National Institutes of Health/National Center for Research Resources, General Clinical Research Center und/oder des Clinical and Translational Science Award unterstützt. | PRO: vom Institutional Review Board genehmigt  Alle Probanden vermuteten bei der Nachuntersuchung nach 3 und 10 Wochen, dass sie echte Akupunktur erhielten  Einbezug von Confounder in  Analyse  Um Verblindung zu  gewährleisten, wurde kein  Feedback bezüglich Qi  Gefühl gefordert  Echte Akupunkturnadeln  wurden auch mit Röhrchen  versehen, was die  Verblindung gewährleisten  sollte  CONTRA:  Keine ITT  Kleine Stichprobe  (Poweranalyse kalkuliert A n=30 und B n=19)  Von den zwei Akupunkteuren  war einer der Autor des  Artikels  Grafische Darstellung der  Ergebnisse, nur p-Werte der  ANOVAs gegeben, keine  Mittelwerte/Mediane/p-Werte  zu einzelnen Zeitpunkten  außer T0  Womöglich keine echte  Akupunktur da die Nadeln  auch mit Röhrchen versehen  wurden | 1b |

| Referenz | Studientyp | Patienten-merkmale | Intervention/  Kontrolle, Beobachtung | Untersuchte Endpunkte | Hauptergebnisse | Neben-/ Wechselwir­kungen | Finanzierung/ Interessens-konflikte | Methodische Bemerkungen | Evidenz-klasse (Oxford) |
| --- | --- | --- | --- | --- | --- | --- | --- | --- | --- |
| Cheng (2017):  Acupuncture for cancer-related fatigue in lung cancer patients: a randomized, double blind, placebo-controlled pilot trial Supportive care in cancer  [2] | RCT  Prospektiv  Monozentrisch  Doppelt verblindet (Pat.)  2 Arme  Eingeschlossen: N=28  Ausgewertet: N= 28  Drop-out: keine  Land: China/Shanghai  2013-2014 (Auswählen der Patienten) | Alter(MW, Range):  60 (18-75)  Geschlecht: 53,6% weiblich  Patienten mit Nicht kleinzelligem Lungenkarzinom Diagnose, die Chemo/Radiotherapie unternommen haben (nicht klar ob bestehend)  Keine Akupunktur zuvor  BFI-C score ≥4  Etc. | **Arm A:**  N=14  Manuelle Körper-akupunktur  4 Wochen, 2x wöchentlich, „de qi“ Nadel Empfinden;  Akupunkturpunkte  LI-4, Ren-6, St-  36, KI-3, and Sp-6;  Tiefe der  Einstiche variiert  von 0.5–  1.5 cun  **Arm B:**  N=14 (Sham Akupunktur Nadeln)  Akupunkteur:  Ausgebildet und 5  Jahre Erfahrung  machte beide  Interventionen | T0: Baseline (Woche -2)  T1: Woche 0  T2: Woche 2  T3: Woche 4  **Primäre Endpunkte:**  1. BFI-C (Chinesische Version des Brief Fatigue Inventory,)  (Skala von 10 für das normale Fatigue Level der letzten 24 Stunden)  **Sekundäre Endpunkte:**  2. FACT-LCS (Functional Assessment of Cancer Therapy-Lung Cancer Subscale)(0-4 Skala) Subjektiv von Patieten und objektiv von Mitarbeitern | **Zu 1.**  mean (SD):  T0: 6.2(A); 6.6(B)  p=0.35  T1: 5.1(A); 6.3(B)  p<0.01  T2: 5.2(A); 6.6(B)  p=0.005  T3: 4.5(A); 7.1(B)  p<0.001  **Zu 2.**  mean (SD):  T0: 86.4(A); 82.1(B)  p=0.23  T1: 93.8(A); 88.5(B)  p=0.11  T2: 96.1(A); 90.3(B)  p=0.04  T3: 98.0(A); 89.3(B)  P=0.002 | Beurteilt mit CTCAE  Alle Nebenwirkungen traten in der echten Akupunkturgruppe auf  Schmerzen: 3 Patienten Grad 1 von 4, Ein Patient Grad 2  Schwindel: 2 Patienten Grad 1 von 4  Bluten: 1 Patient Grad 1 von 4  => Bei keinem der Probanden traten schwerwiegende Nebenwirkungen auf | Diese Studie wurde durch Zuschüsse des Comprehensive and Integrative Medicine Institute (CIMI) und der National Natural Science Foundation of China gefördert  Laut  Autoren  keine COI | PRO:  Ethikvotum  Kleine Stichprobe Statistisch berücksichtigt, allerdings nicht erklärt wie genau  Glaubwürdigkeits Fragebögen für Blindungsüberprüfung  Intention-to-treat (ITT) population  Akupunktur nach STRICTA  protokolliert (Standards für die  Berichterstattung über Interventionen in klinischen  Studien zur Akupunktur)  CONTRA:  Kleine Stichprobe (28) – keine  Poweranalyse  Alle Interventionen von einem Akupunkteur ausgeführt-> könnte Einfluss auf Patienten gehabt haben  Mögliche Beeinflussung durch Finanzierung | 1b |

| Referenz | Studientyp | Patienten-merkmale | Intervention/  Kontrolle, Beobachtung | Untersuchte Endpunkte | Hauptergebnisse | Neben-/ Wechselwir­kungen | Finanzierung/ Interessens-konflikte | Methodische Bemerkungen | Evidenz-klasse (Oxford) |
| --- | --- | --- | --- | --- | --- | --- | --- | --- | --- |
| Deng (2013): Acupuncture for the treatment of post-chemotherapy chronic fatigue: a randomized, blinded, sham-controlled trial. Support Care Cancer. [3] | RCT  Prospektiv  Monozentrisch  Einfach verblindet (Pat.)  2 Arme  Eingeschlossen: N=101(123)  Ausgewertet: N=97  Drop-Out:  A/B n=15/11 (n=2/1 vor Intervention, n=2/1 Studie verlassen, n= 4/3 Lost to Follow-up, n=7/7 Andere (Gründe nicht erläutert))  Land: USA  2004-2009  (Erhebung, Rekrutierung von MSKCC Patienten) | Alter(MW, Range):  53 (18–64) Jahre  Geschlecht: 82% weiblich  Gemischte Krebsdiagnosen, Post- Chemotherapie (mind. 60 Tage zuvor), laufende OPs, Immun-/Radiotherapie; Hormontherapie/Opiat med. Änderungen vor weniger als 3 Wochen startend (2% mit fortlaufender Antihormontherapie); keine Akupunktur/SSRI med. Änderungen innerhalb der letzten 6 Wochen  Hospital Anxiety and Depression Scale (HADS) >11 | **Arm A:**  N=49  Manuelle Körper-akupunktur  6 Wochen, 1x wöchentlich + UC, „de qi“ Nadel Empfinden  Folgende Akupunkturpunkte wurden ausgewählt: GV20, Ex-HN3, HT7, PC6, ST36, SP6, KI3, LR3 und Ear Shen Men  **Arm B:**  N=52 (Sham Akupunktur Nadeln (stumpf) und Punkte, die wenige Millimeter von den Meridianpunkten der echten Akupunktur entfernt) +UC  In Woche 7 Möglichkeit echte Akupunktur zu erhalten | Baseline T0: Durchschnitt aus Messungen an Tag -14 und -7 vor erster Akupunktursitzung  Unterschied nach der Behandlung T1:  Durchschnitt aus Tag 42 und Tag 49  **Primäre Endpunkte:**  1. Fatigue (BFI, Tag -14, -7, 42, 49, 180)  **Sekundäre Endpunkte:**  2. Lebensqualität (tumorspezifisch, FACT-G, Tag -14, -7)  3. Ängstlichkeit (HADS, Tag -14, 42)  4. Depressivität (HADS, Tag -14, 42)  Zusatz: BFI nach 6 Monaten, nur Akupunktur | Zu 1. Unterschied nach der Behandlung: A vs. B kein  sign. Unterschied  (Post-treatment difference, SD adjusted for baseline scores): 0.04 (95 % KI  −0.57, 0.66); p=0.9,  adjustiert für  Baselinewerte)  Zu 2. FACT-G (insgesamt): 0.10 (95%CI-3.37,3.57) p=1.0  Zu 3.:  -0.21  (95%CI-1.35,0.93)  P=0.7  Zu 4.: -0.10 (95%CI-1.61,1.41) p=0.9  -> **A vs B:**  Post Intervention:  Keine sign. Gruppenunterschiede (p≥0.05)  **Zusatz: BFI nach 6 Monaten** (n= 36 (A) + 28 (von B gewechselte) = 64)  -> keine sign. Unterschiede (p=0.7) | SUEs: 11  kein SUE schien mit Akupunktur zusammenhängend, keine weiteren NW/WW berichtet | Keine Angaben zur Finanzierung  Laut Angaben kein COI | PRO:  ITT Analyse  Adjustierung für Baseline-Charakteristika (Alter, Geschlecht, Ausgangswert Müdigkeit/Depression/Angst/Hgb)  Gruppengröße>30  Randomisierung stratifiziert für Baseline BFI>6  Poweranalyse  Prüfen der Verblindung  CONTRA:  Wenig Info in Tabelle gegeben  Viele, unbegründete Drop-outs  Keine genauen Werte gegeben | 1b |

| Referenz | Studientyp | Patienten-merkmale | Intervention/  Kontrolle, Beobachtung | Untersuchte Endpunkte | Hauptergebnisse | Neben-/ Wechselwir­kungen | Finanzierung/ Interessens-konflikte | Methodische Bemerkungen | Evidenz-klasse (Oxford) |
| --- | --- | --- | --- | --- | --- | --- | --- | --- | --- |
| Du (2021)  Prevention and treatment of acupuncture for cancer-related fatigue caused by chemotherapy of intestinal cancer: A randomized controlled trial  World Journal of Acupuncture | RCT  Prospektiv  Monozentrisch  Offen  2 Arme  Eingeschlossen: N=61  Ausgewertet: N=50  Drop-out: 11 (rein mathematisch erschlossen. Aus der Studie gehen keine Gründe hervor)  Land: China/ Guangdong Provincial Hospital of Chinese Medicine  Juni 2018 bis Januar 2019 | Alter(Range):  (18-80)  Geschlecht: 34% weiblich  Patienten mit Kolorektalkarzinom Diagnose, die Chemotherapie unternommen haben  Alle Patienten in den beiden Gruppen erhalten FOLFOX und FOLFIRI  oder XELOX-Chemotherapie | **Arm A:**  N=26  Manuelle Körper-akupunktur + Chemotherapie  (8 Behandlungen mit Akupunktur: ein Tag vor der Chemotherapie und vom 1. bis zum 3. Tag der Chemotherapie separat einmal täglich für jeweils 30 Minuten. Insgesamt zwei Zyklen, jeweils 3 Wochen); bilateral ST36, CV6 and CV4  **Arm B:**  N=24  Nur Chemotherapie  (2 x 3Wochen) | T0: Vor Behandlungsstart  (keine genaue Zeitangabe)  T1: 6 Wochen nach Behandlungsstart bzw. 3 Wochen nach letzter Akupunkturtherapie  **Primäre Endpunkte:**  1. PFS (Verhalten, Emotionen, Wahrnehmung, Erkenntnis)  PS score  **Sekundäre Endpunkte:**  2. EORTC-QLQ-C30 (Körperlich, Rolle, Emotional, Kognitiv, Sozial, Müdigkeit, Dyspnoe) | **Zu 1.:**  Arm A vs B  T0:  Mittelwerte aller Dimensionen und Gesamtscore: t = -0.697, 0.179, Z = -1.089, t = -2.014, -1.191, p = 0.489, 0.859, 0.276, 0.050, 0.240, > 0.05  -> keine signifikanten Unterschiede  T1:  Mittelwert der Wahrnehmung: t = 2,465, p = 0,019, < 0,05  -> In Arm A signifikant verringert  Mittelwerten von Verhalten, Emotion und Vorstellung, sowie des Gesamtwertes: Z = -1,672, -1,168, -0,116, 1,708, p = 0,095, 0,243, 0,908 , 0,096, > 0,05  -> nicht signifikant  T0 vs T1:  Arm A(Mittelwerte von Verhalten, Emotion und Konzeption sowie den Gesamtscore): t = 4.947, 2.371, 4.687, 2.311, 7.559, p < 0.001, 0.026, < 0.001, 0.029, < 0.001  -> alle signifikant reduziert  Arm B(Mittelwerte von Verhalten, Emotion, Vorstellung und des Gesamtwertes): t = 1.294, 1.571, -0.328, -0.259, 0.859, p = 0.209, 0.130, 0.746, 0.798, 0.399, > 0.05  -> kein statistisch signifikanter Unterschied  **Zu 2.:**  Arm A vs. B:  T0:  Mittelwert des emotionalen Feldes. Z = -2.443, p = 0.015  -> signifikanter Unterschied zwischen den beiden Gruppen, daher keine vergleichbare Baseline  Mittelwerte der körperlichen-, rollen-, kognitiven- und sozialen Funktion: t = -0.063, 1.529, Z = -0.558, -0.304, p = 0.950, 0.133, 0.577, 0.761, > 0.05  -> kein sign. Unterschied also konsistente/vergleichbare Baseline  Mittelwerte für den allgemeinen Gesundheitszustand und jedes Symptomfeld (außer „Verstopfung“)-> kein sign. Unterschied, also konstante/vergleichbare Baseline  T1:  Mittelwert der körperlichen Funktion: Z = -2.233, p = 0.026, < 0.05  -> signifikant erhöht in Arm A  Mittelwerte für Rolle, kognitive und soziale Funktionen: Z = -0.042, t = -0.837, Z = -0.856, p = 0.966, 0.408, 0.392, > 0.05  -> keine sign. Unterschiede  Mittelwerte für den allgemeinen Gesundheitszustand und jedes Symptomfeld: Z = 2.475, t = 3.001, Z = -2.537, p = 0.013, 0.004, 0.011, < 0.05  -> sign. Reduziert für Arm A  T0 vs T1:  Arm A:  Mittelwert körperlicher-, rollen- und sozialer Funktionen: t = 4.560, Z = -3.279, 2.951, p = 0.000, 0.001, 0.003  -> sign. Unterschied  Mittelwert der kognitiven Funktion: Z = -0.957, p = 0.339  -> kein sign. Unterschied  Mittelwerte für allgemeine Gesundheit, Müdigkeit und Atemnot: Z = -2.993, 3.828, -2.121, p = 0.003, 0.000, 0.034  -> sign. Unterschied  Arm B:  Mittelwerte der körperliche-, rollen-, kognitive- und soziale Funktionen: Z = -0.566, t = 0.327,Z = 0.615,t = -1.219, p = 0.571,0.746,0.539,0.223  -> kein sign. Unterschied  Mittelwerte für allgemeine Gesundheit, Müdigkeit und Atemnot: t = 1.741, 0.041, Z = 0.0 0 0, p = 0.095, 0.968, 1.0 0 0  -> Kein sign. Unterschied | Bei den Patienten der Behandlungsgruppe kam es während des Eingriffs mit Akupunktur zu keinen unerwünschten Ereignissen (z. B. Ohnmachtsanfälle, Allergien, Infektionen und organische Verletzungen) | Keine Angaben zur Finanzierung | PRO: Ethikrat bewilligt  Shapiro-Wilk test  CONTRA:  Drop-outs unklar  Keine genauen Daten zum Zeitpunkt der Resultaterhebungen  Keine Vergleichbarkeit im emotionalen Feld (Z = -2.443, p = 0.015).  Keine Verblindung, keine Sham  Akupunktur –  Aufmerksamkeitseffekte können  nicht ausgeschlossen werden  Keine Poweranalyse/ITT | 1b |

| Referenz | Studientyp | Patienten-merkmale | Intervention/  Kontrolle, Beobachtung | Untersuchte Endpunkte | Hauptergebnisse | Neben-/ Wechselwir­kungen | Finanzierung/ Interessens-konflikte | Methodische Bemerkungen | Evidenz-klasse (Oxford) |
| --- | --- | --- | --- | --- | --- | --- | --- | --- | --- |
| Li (2020)  ATAS Acupuncture Reduces Chemotherapy Induced Fatigue in Breast Cancer Through Regulating ADROA1 Expression: A Randomized Sham-Controlled Pilot Trial  OncoTargets and therapy | RCT  Prospektiv  Monozentrisch  Verblindet (für sham and ATAS, nicht für C)  Für alle randomisiert  3 Arme  Eingeschlossen: N=40  Ausgewertet: N=37  Drop-Out:  A/B/C n=2/0/1 (n=1/0/0 konnte zu wenig Akupunkturen absolvieren aufgrund der Anreisedistanz, n=1/0/0 Fernmetastasen entdeckt, n=0/0/1 wollte Akupunktur)  Land: China, Kunming center  2018 (Erhebung, Rekrutierung von BRCA Patienten) | Alter(MW, Range):  47.5/42/50.5 (18+) Jahre  Geschlecht: 100% weiblich  BRCA Stadium 0-3 (kürzlich diagnostizierter oder operierter primärer Brustkrebs)  ohne Hinweis auf Fernmetastasen.  Patienten mit geplanter Chemotherapie (4 Zyklen EC4P4, gefolgt von 4 Zyklen Paclitaxel als Standardprotokoll).  KPS>60  Keine bekannten schweren Entzündungs- oder Stoffwechselkrankheiten oder andere unkontrollierte medizinische Zustände oder Komorbiditäten, die die Teilnahme des Probanden beeinträchtigen könnten  Keine Akupunktur in den letzten 4 Monaten | **Arm A:**  N=20 18  Adjuvante Chemotherapie (Epirubicin und Cyclophosphamid, gefolgt von Paclitaxel (Epirubicin 90 mg/m2 plus Cyclophosphamid 600 mg/m2, alle 3 Wochen x4, gefolgt von Paclitaxel 175 mg/m2, alle zwei Wochen x4))  +  ATAS Akupunktur (Acupoints Time-Space Acupuncture)  **Arm B:**  N=10  Adjuvante Chemotherapie  +  Sham Akupunktur (Feine und kurze Nadeln zum minimalinvasiven, flachen Nadeleinstich an Nicht-Akupunkturpunkten)  ATAS/Sham: 20 Wochen, 1 mal pro Woche für 60 min.  Nadeln und Nadel stehen lassen für je 30 Minuten  **Arm C:**  N=10 9  Nur adjuvante Chemotherapie | T0: Baseline (Zeitpunkt der Randomisierung)  T1: nach 3-4 EC Zyklen  T2:nach 3-4 Paclitaxel Zyklen  T3: 1 Monat post- Chemotherapie 🡪 ausgeschlossen von Analyse  **Primäre Endpunkte:**  Fatigue:  1. VAS-F  Wöchentlich erhoben + T3  2. MFI-20  T 0,1,2, 3  **Sekundäre Endpunkte:**  3. Ängstlichkeit und Depressivität mit  Hospital Anxiety and Depression Scale (HADS)  Schlafqualität T 0,1,2, 3  4. Insomnia Severity Index (ISI) T 0,1,2, 3 | Mean scores  Zu 1.:  T0:  A=1.3±1.2  B=1.3±1.0  C=2.1±1.5  F=1.547  P=0.229  Verringerte sich um 0.351 bzw. 1.198 in Arm A im Vergleich zu Arm B und C.  A vs. B/C: p = 0.004.  Signifikante vergleichsweise Verbesserung ab Woche 7 (Beginn 3. Zyklus Chemotherapie)  Zu 2.:  T0:  A=46.4±9.1  B=39.3±12.5  C= 45.8±15.7  F=1.031  p=0.368  T2: B/C = 49.0±12.8/56.8±18.7 (am höchsten)  A = 43.1±18.1 (fällt)  Over time:  A: p=0.016  B/C: p=0.028  T3: B/C = niedriger  A: almost maintained  Zu 3.:  T0:  A=10.2±6.1  B=8.3±6.1  C=9.4±3.9  F=1.072  p=0.354  B vs. C = B um 1.380 verringert  A vs. B/C = A um 0.513 und 1.893;  n.s. verringert  Zu 4.:  T0:  A=4.5±3.5  B=3.0±3.7  C=5.6±4.4  F=0.739  p=0.485  T0-T1: B/C steigend, dann sinkend in A/C.  T1-T2: am niedrigsten für Arm A; sank um 0,874 bzw. 0,630 im Vergleich zu B/C. | Wenn die Teilnehmer nach der Akupunktur nicht richtig auf die Akupunkturpunkte drückten, traten bläuliche Blutergüsse auf, die nach ein bis zwei Tagen von selbst verschwanden.  Sonst keine Nebenwirkungen | staatliche Förderung. Die Förderagenturen waren laut Studie weder an der Konzeption und Durchführung der Studie noch an der Erhebung, Verwaltung, Analyse, Interpretation der Daten und Verfassen des Manuskripts beteiligt | PRO:  Intention to treat Analyse (Primäre Endpunkte)  per-protocol basis (weniger als 75% der Interventionen abgeschlossen wurden ausgeschlossen)  Ethikkommission genehmigt  Gruppengröße>30 (Pilotstudie)  Prüfung der Verblindung (Fragen der Gruppen A und B, alle glaubten wären in A)  CONTRA:  Gefördert  Keine tabellarische Übersicht der Ergebnisse und Messzahlen,  Ergebnisse schwer nachvollziehbar durch fehlende Werte zu den Zeitpunkten und wenig Information zu statistischen Unterschieden. | 1b |

| Referenz | Studientyp | Patienten-merkmale | Intervention/  Kontrolle, Beobachtung | Untersuchte Endpunkte | Hauptergebnisse | Neben-/ Wechselwir­kungen | Finanzierung/ Interessens-konflikte | Methodische Bemerkungen | Evidenz-klasse (Oxford) |
| --- | --- | --- | --- | --- | --- | --- | --- | --- | --- |
| Li (2023)  Effects of Mind-Regulation Acupuncture Therapy on Serum Ghrelin, Gastric Inhibitory Polypeptide, Leptin, and Insulin Levels in Breast Cancer Survivors with Cancer-Related Fatigue: A Randomized Controlled Trial  International journal of general medicine | RCT  Prospektiv  Monozentrisch  Blind für Therapeuten und  Auswerter  2 Arme  Eingeschlossen: N=136  Ausgewertet: N=114  Drop-Out:  A/B n=11/11 (n=4/0 andere Therapie die das Ergebnis beeinflussen hätte können, n=7/4 unzureichende Behandlung, n= 0/1 Schwangerschaft, n=0/6 Erhalt von Schlafmitteln)  Land: China, Huzhou Hospital of Traditional Chinese Medicine  2020 - 2021 (Erhebung, Rekrutierung von BRCA Patienten) | Alter(MW, Range):  53 (18–75) Jahre  Geschlecht: 100% weiblich  BRCA Stadium 0-3  Hatten eine Strahlentherapie oder Chemotherapie, hatten ein Jahr zuvor eine BRCA-Operation und waren während dieser Studie keiner Chemotherapie oder Strahlentherapie ausgesetzt und waren zum Zeitpunkt der Aufnahme frei von Krebsmetastasen und Rezidiven.  Erfüllen diagnostische Kriterien für CRF.  KPS-Wert >= 60  Haben in den letzten sechs Monaten keine Akupunktur erhalten haben | **Arm A:**  N=57  Selbe Behandlung wie die Kontrollgruppe + MRA (mind regulating acupuncture)  4 Wochen, 5 mal pro Woche, Nadeln in 10 min. Abständen für 30 min.)  Mit leicht verstärkender und abschwächender Weise gestochen, bis eine nadelnde Reaktion erreicht wird  Folgende Akupunkturpunkte wurden ausgewählt: Baihu (GV20), Yintang (GV29), Taichong (LR3), Hegu (LI4), Zusanli (ST36), Sanyinjiao (SP6), and Shenmen (HT7)  (0.30 mm × 50 mm, Dong Bang Brand, sterile Nadeln)  **Arm B:**  N=57 psychologische, gesundheitliche und sportliche Beratung sowie Ernährungsunterstützung, basierend auf ihrem Allgemeinzustand. intravenöse Injektion von A (250 ml, einmal täglich und fünfmal pro Woche für vier aufeinanderfolgende Wochen; ein Behandlungszyklus | T0: Vor der Behandlung (Behandlungsstart)  T1: Nach der Behandlung (keine genauen Zeitangaben)  **Primäre Endpunkte:**  Fatigue:  1. MFI-20  **Sekundäre Endpunkte:**  Serum Metabolit Levels anhand von Blood(EDTA);  Electrochemiluminescence immunoassay:  2. Serum insulin  ELISA:  3. Ghrelin  4. Gastric inhibitory polypeptide (GIP)  5. Leptin  Schlafqualität  6. Pittsburgh Sleep Quality Index (PSQI)  Depressions Grad  7. Hamilton Depression Scale (HAMD)  Lebensqualität  8. KPS scale | T-Test zum Vergleich von A/B für Daten mit Normalverteilung  Mann-Whitney-U-Test für Daten, die nicht einer Normalverteilung entsprechen  Die Zähldaten wurden mit dem Chi-Quadrat-Test oder dem exakten Fisher-Test getestet  statistischer Unterschied bei P <0,05  Zu 1.:  Gesamtergebnisse:  T0:  A: 73.05 (+/- 4.07)  B:71.61 (+/- 4.85)  T1:  A:40.74 (+/- 3.54)  B: 53.07 (+/- 4.2)  Signifikant stärkere Verbesserung in Arm A (p<0.05)  Zu 2.  T0:  A: 506.13 ± 74.97  B: 490.46 ± 64.36  Kein sign. Unterschied zwischen A und B (p>0.05)  T1:  A: 247.35 ± 73.97  B: 341.37 ± 86.84  Signifikant stärker reduziert in Arm A vs. B (p<0.01)  Zu 3.:  T0:  A: 372.36 ± 37.37  B: 373.20 ± 31.94  Kein sign. Unterschied zwischen A und B (p>0.05)  T1:  A: 207.07 ± 24.03  B: 292.14 ± 22.03  Signifikant stärker reduziert in Arm A  Signifikant stärker reduziert in Arm A vs. B (p<0.01)  Zu 4.:  T0:  A: 213.18 ± 32.54  B: 210.57 ± 33.92  Kein sign. Unterschied zwischen A und B (p>0.05)  T1:  A: 95.71 ± 16.99  B: 140.19 ± 22.60  Signifikant stärker reduziert in Arm A  Signifikant stärker reduziert in Arm A vs. B (p<0.01)  Zu 5.:  T0:  A: 12.73 ± 2.27  B: 3.10 ± 1.84  Kein sign. Unterschied zwischen A und B (p>0.05)  T1:  A: 5.76 ± 1.49  B: 9.75 ± 1.64  Signifikant stärker reduziert in Arm A  Signifikant stärker reduziert in Arm A vs. B (p<0.01)  Zu 6.:  Gesamtergebnisse:  T0:  A: 2.53 ± 3.22  B: 11.84 ± 3.99  Keine sign. Unterschiede (P>0.05) A vs. B  T1:  A: 5.86 ± 2.26  B: 7.95± 2.68  Schlafqualität, Schlaflatenz, Schlafdauer, Schlafeffizienz, Anwendung hypnotischer Arzneimittel und die gesamten PSQI-Werte T1: Signifikant mehr verringert in Arm A verglichen zu Arm B.  (p < 0.05 oder p  < 0.01); n.s. für Schlafstörungen nach der Behandlung und Funktionsstörungen am Tag  Zu7.:  T0:  A: 33.23 ± 5.45  B: 34.14 ± 4.68  A vs. B keine nennenswerten Unterschiede (p>0,05)  T1:  A: 19.11 ± 4.52  B: 21.65 ± 5.21  A/B: deutliche Abnahme (p<0,01)  Größere Verbesserung in Arm A im Vergleich zu B)  Zu 8.:  T0:  A: 74.04 ± 7.04  B: 72.28 ± 8.46  A vs. B keine nennenswerten Unterschiede (p>0,05)  T1:  A: 50.18 ± 8.13  B: 56.58 ± 8.62  A/B: deutliche Zunahme (p<0,01)  Größere  Verbesserung in A  im Vergleich zu B) | Keine Informationen  Laut  Autoren  Kein Interessenkonflikt | Natural Science Foundation of Zhejiang Province | PRO:  Für dieses Werk wurden von den Autoren keine konkurrierenden Interessen angegeben  Ethikkommission genehmigt  Gruppengröße>30  CONTRA:  Keine ITT Analyse  Gefördert  kleine Stichprobengröße  kurze Beobachtungszeit  kurze Nachlaufzeit  Keine leere Kontrollgruppe oder Scheinakupunkturgruppe  kein Multicenter etabliert | 1b |

| Referenz | Studientyp | Patienten-merkmale | Intervention/  Kontrolle, Beobachtung | Untersuchte Endpunkte | Hauptergebnisse | Neben-/ Wechselwir­kungen | Finanzierung/ Interessens-konflikte | Methodische Bemerkungen | Evidenz-klasse (Oxford) |
| --- | --- | --- | --- | --- | --- | --- | --- | --- | --- |
| Eonju (2019):  Acupuncture for the Treatment of Fatigue after Total Thyroidectomy in Women with Papillary Thyroid Cancer: A Pilot Study  Acupuncture & Electro-Therapeutics Research 2019 | RCT  Prospektiv  Monozentrisch  Offen  2 Arme  Eingeschlossen: N=26  Ausgewertet:  N=25  Drop-out: A/B n = 1/0 (hatte nach einer Akupunkturbehandlung Kopfschmerzen und Herzklopfen)  Land : Korea,  Department of Internal Medicine Catholic University of Daegu School of medicine, Daegu Juli bis Dezember 2013 an einem tertiären Lehrkrankenhaus | Patienten mit PTC, die mehr als 3 Monate nach der totalen Thyreoidektomie über Gewichtszunahme oder Müdigkeit berichteten  Alter (MW A/B):  49.75+-8.82/44.85+-5.27  Geschlecht: 100% weiblich | **Arm A:**  N=12  Manuelle Körper-akupunktur  6 Wochen, 2 x wöchentlich für 25-30 min.  Saam Akupunktur  Akupunkturpunkte: Um den Mangel an Milzmeridianen auszugleichen, wurden SP-1, SP-2, HT-8 und LR-1 an der rechten Extremität verwendet. Um die Kälte des Nierenmeridians auszugleichen, wurden HT-3, HT-8, KI-2 und KI-10 an der linken Extremität verwendet. Zusätzlich zu den Saam-Akupunkturpunkten wurden auch ST-9, ST-10, ST-36 (bilateral), CV-4, CV-6 und CV-22 verwendet. Während jeder Akupunktursitzung erhielt jeder Patient insgesamt 17 Akupunkturnadeln. Die Auswahl dieser Akupunkturpunkte basierte auf einem Konsens zwischen dem koreanischen Arzt der Studie und Professoren des College of Korean Medicine der Daegu Haany University  Nadeln: 0.20mm Durchmesser, 40mm Länge  10–20 mm tief in die Haut ein und wurden sanft manipuliert, um De-Qi zu erhalten.  **Arm B:**  N=13 Kontrollgruppe | T0: Baseline  T1: nach 12 Behandlungssitzungen  **Primäre Endpunkte:**  Fatigue  1. Fatigue severity scale (FSS)  Lebensqualität  2. Short Form-36 (SF-36)  **Sekundäre Endpunkte:**  3. Gewicht, BMI und Dicke der Trizepsfalte, Glukose-, Insulin- und Lipidprofile, (HOMA-IR) Schilddrüsenfunktionstests, Thyreoglobulin (Tg) und Anti-Tg-Antikörper, Serum-Aspartat-Aminotransferase (AST), Alanin-Aminotransferase (ALT) und Gesamtbilirubin | Zu 1.: MW (SD)  A T0: 29.92 (13.111)  Zu T1: 19.50 (10.536);  B T0: 29.92 (13.029) zu T1: 31.15 (12.877);  Veränderung über die Zeit sign. verschieden (Zeit*Gruppe) p=0.011;  In A verringert 91,7 %, erhöht 8,3 %;  In B verringert; 46,2 %, keine Veränderung; 7,6 %, erhöht; 46,2 %). 11/12 in A (91,7 %) verbesserten den FSS-Score signifikant;  Zu 2.  A 74.12 vs. B 69.02 nicht sign.; p=0.085  Zu 3. keine sign. Unterschiede | Laut Autoren kam es bei keinem der Probanden zu Nebenwirkungen (bis auf Drop-Out) | Zuschuss vom Ministerium für Gesundheit und Soziales der Republik Korea. | PRO:  Ethikvotum  Ein Akupunkteur  CONTRA:  Keine ITT  kleine Stichprobe  Obwohl die Wahrscheinlichkeit, an Schilddrüsenkrebs zu erkranken, bei Frauen zwei- bis dreimal höher ist als bei Männern, wurden nur Frauen mit PTC in die Studie aufgenommen  Die Nachbeobachtungszeit nach der Behandlung war kurz  Akupunkturpunkte wurden individuell bestimmt 🡪 keine Vergleichbarkeit | 1b |

| Referenz | Studientyp | Patienten-merkmale | Intervention/  Kontrolle, Beobachtung | Untersuchte Endpunkte | Hauptergebnisse | Neben-/ Wechselwir­kungen | Finanzierung/ Interessens-konflikte | Methodische Bemerkungen | Evidenz-klasse (Oxford) |
| --- | --- | --- | --- | --- | --- | --- | --- | --- | --- |
| Molassiotis (2012): Acupuncture for cancer-related fatigue in patients with breast cancer: a pragmatic randomized controlled trial.  J Clin Oncol. [9] | RCT  Prospektiv  Bizentrisch  offen  2 Arme  Eingeschlossen: N=302  Ausgewertet:  N=246  Drop-Out:  A/B n=46/10  (haben die Endergebnisse nicht abgeliefert)  Arm A: 9 ohne Intervention: davon 6 selbst ausgeschlossen,3 vom Forscher ausgeschlossen;  53 mit weniger als 6 Sitzungen: davon 52 selbst, 1 vom Forscher)  Land: Groß­britannien (Spezialkliniken für Krebserkrankungen)  Zeitraum nicht berichtet | Einschluss:  Mamma-Karzinom (Stadium I bis IIIA)  nach adjuvanter Therapie (5 Jahre bis minimal ein Monat zuvor)  Chronische Fatigue (10-point scale score >= 5)  Patienten mit einem früheren Lokalrezidiv waren teilnahmeberechtigt, nicht jedoch Patienten mit Fernmetastasen | Arm A:  N=227 181  Manuelle Körper Akupunktur für 20 Minuten, bei der beidseitig oder einseitig drei Punkte (ST36, SP6 und LI4) genadelt wurden, für den Fall, dass die Punkte nicht punktiert werden konnten (z. B. im Falle eines Lymphödems), alternative Punkte (von den Therapeuten nach eigenem Ermessen ausgewählt): zBsp. GB34 und SP9.  6 Wochen, 1x wöchentlich  + Übliche Betreuung (+ Fatique Interventions Broschüre)  Die Punkte wurden senkrecht und mit einer Tiefe von 0,5 bis 1 Zoll durchstochen  Arm B:  N=75 65 Übliche Betreuung (+ Fatique Interventions Broschüre) | T0: Baseline  T1: Nach der Behandlung (6 Wochen)  Primärer Endpunkt:  1. Fatigue (MFI)  Sekundäre Endpunkte:  2. Ängstlichkeit (HADS)  3. Depressivität (HADS)  4. Lebensqualität (tumorspezifisch) (FACT-B) | 1. Fatigue:  Zu Woche 6: sign. Unterschied zwischen A und B (p< 0,001), zugunsten A  konservative Sensitivitätsanalyse für Drop-outs: Effekt von A reduziert: -2,49(95%CI, -3,29 bis -1,69); immer noch sign. (p<0,001)  2./3./4. Ängstlichkeit/ Depressivität/Lebens­qualität (Tumor spezifisch):  → Akupunktur + UC  versus UC: Post Intervention:  signifikante Gruppen Unterschiede zugunsten Akupunktur (p<0.05) | Keine Angabe zu SUEs/UEs. | Break­through Breast Cancer  (Großbri­tanien)  Laut Angaben kein COI | PRO:  Ethikvotum  ITT Analyse  ethische Genehmigung durch eine Forschungsethikkommission  und alle beteiligten Krankenhäuser und Zentren  Überwachung unerwünschter Ereignisse anhand von Patientenberichten und der Durchsicht der Aufzeichnungen der Therapeuten.  Gruppengröße  Erhebung von KAM Nutzung vor und während der Studie  Poweranalyse  Sensitivitätsanalyse (Ansatz des letzten Wertvortrags)  Kein Unterschied für Einbezug von Fatigue Score oder Hormontherapie Status  Die Gespräche zwischen Akupunkteuren und Patienten wurden auf ein Minimum beschränkt  CONTRA:  Keine Mittelwerte und genaue p-Werte oder andere statistische Werte angeben, nur Vergleichswerte  Offenes Design  Die durchschnittliche Dauer der Fatigue betrug 18 Monate (Bereich: 4 bis 58 Monate) im Standardpflegearm und 15 Monate (Bereich: 2 bis 69 Monate) im Akupunkturarm -> möglicher bias in der Vorauswahl  Keine klaren Baseline Vergleichswerte  Die Follow-up Daten (Woche 10 und 18) sind nicht berichtet – Verweis auf eigenständige Publikation  Übliche Betreuung ist nicht einheitlich, zudem sehr passiv (kein Ansprechpartner für B)  Möglicher Bias durch Besuch von A im Krankenhaus, während B zuhause ist  Keine Angabe der Baselinewerte für sekundäre Endpunkte  Keine genauen Werte wie Mittelwerte zu Endpunkten geben, deshalb ist Änderung über die Zeit schwer nachvollziehbar  Angabe der Baseline Fatigue nur in Kategorien oder grafisch, Mittelwert für B scheint leiht höher zu sein, jedoch nicht ablesbar | 1b |

| Referenz | Studientyp | Patienten-merkmale | Intervention/  Kontrolle, Beobachtung | Untersuchte Endpunkte | Hauptergebnisse | Neben-/ Wechselwir­kungen | Finanzierung/ Interessens-konflikte | Methodische Bemerkungen | Evidenz-klasse (Oxford) |
| --- | --- | --- | --- | --- | --- | --- | --- | --- | --- |
| Molassiotis (2013)  A randomized, controlled trial of acupuncture self-needling as maintenance therapy for cancer-related fatigue after therapist-delivered acupuncture  Annals of oncology | RCT  Prospektiv  Monozentrisch  Unverblindet  3 Arme  Eingeschlossen: N=197  Ausgewertet: N=151  Drop-Out:  A/B/C n=16/9/21 (Haben T0/T1 Enddaten nicht übermittelt))  Land: UK, 2012  (Erhebung, Rekrutierung von BRCA Patienten) | Alter(MW, Range):  53 Jahre  Geschlecht: 100% weiblich  Diagnose Brustkrebs im Stadium I, II oder IIIa  Abgeschlossene Chemotherapie vor mindestens 1 Monat (durchschnittliche Zeit von 20 Monaten zuvor).  Drei Viertel der Stichprobe in jedem Arm erhielten eine biologische oder  hormonelle Behandlungen.  Sechswöchentliche Sitzungen zur klinikbasierten Akupunktur als Teil der vorherigen Phase der Studie absolviert  Lebenserwartung > 6 Monate  Rerandomisierung der Akupunktur Erhalter aus anderem Trial | **Arm A:**  N=49  20-Minütige Akupunktursitzung pro Woche für (nach 6 Wochen weitere) 4 Wochen  bilaterale Nadelung von 3 Punkten (ST36, SP6, LI4) von trainiertem Akupunkteur (n = 12)  Nadeln: Seirin mit Führungsrohren zum Einmalgebrauch, 36 gauge/point 16–30 mm  **Arm B:**  N=56  Selbstnadelung für weitere 4 Wochen, wie in ArmA nachdem den Teilnehmern von erfahrenen Akupunkteuren beigebracht wurde, sich selbst zu nadeln  **Arm C:**  N=46  Keine weitere Akupunktur | T0: Baseline (Zeitpunkt der Re-randomisierung = nach 6 Wochen der Ursprungsstudie)  T1: 4 Wochen nach T0 (10 Wochen)  T2: 12 Wochen nach T0 (18 Wochen)  **Primäre Endpunkte:**  Generelle Fatigue:  1. MFI-20 (GF 6 − GF 10) zu T1  **Sekundäre Endpunkte:**  Mentale fatigue, Ängstlichkeit, Depressivität und Lebensqualität  2. Hospital Anxiety and Depression Scale (HADS) zu T1  3. Endpunkte 1 und 2 zu T2 | 1. T0-T1  Mittlere Differenz  A: 0.57 (−0.18 zu 0.04  Vs. B: 0.54 (−0.21 zu 0.13), n.s., p=0.18;  C: (−0.35, −0.52 zu 1.21) vs. A/B (−0.76 , -1.59 to 0.06), n.s., p=0.07;  ANOVA: n.s., p= 0.13  2. Mittlere Differenz zu C Effekt/SE/95% CI zu T1:  Ängstlichkeit:  A: 0.10/0.52/−0.92 zu 1.12  B: −0.50/0.53 /−1.55 zu 0.56  Depressivität:  A: 0.03/0.48/−0.92 zu 0.97  B: −0.20/0.49/−1.16 zu 0.76;  Alle Vergleiche nicht signifikant (kein p-Wert gegeben)  3. Keine sign. Ergebnisse für GF in Behandlungseffekt oder Veränderung über die Zeit in Armen, kein sign. Unterschied in der mittleren Differenz A/B vs. C (0.57 -0.49 zu 1.64; p=0.29); keine sign. Unterschiede zwischen Armen für MFI, HADS und FACT-B für mittlere Differenz zu C | Nebenwirkungen wurden überwacht. Von Patienten in der Selbstakupunkturgruppe wurden keine Nebenwirkungen berichtet (mit Ausnahme einer kleinen Anzahl von Fällen mit punktuellen Blutungen und leichten Schmerzen/Beschwerden) daher wurde dies als sichere Alternative geschlossen | Stipendium von Breakthrough Breast Cancer  Die Autoren haben keine Interessenkonflikte. | PRO:  Ethikvotum  Poweranalyse  STRICA  CONTRA:  Keine ITT  23,35%, hoher Drop out  Folgestudie, daher potentieller Einfluss von voriger Studie  Keine Werte der sekundären zu Arm C, nur Angabe der Vergleiche | 1b |

| Referenz | Studientyp | Patienten-merkmale | Intervention/  Kontrolle, Beobachtung | Untersuchte Endpunkte | Hauptergebnisse | Neben-/ Wechselwir­kungen | Finanzierung/ Interessens-konflikte | Methodische Bemerkungen | Evidenz-klasse (Oxford) |
| --- | --- | --- | --- | --- | --- | --- | --- | --- | --- |
| Smith (2013)  The effect of acupuncture on post-cancer fatigue and well-being for women recovering from breast cancer: a pilot randomised controlled trial  Acupuncture in medicine | RCT  Prospektiv  Monozentrisch  Blind für Arm A/B und  Assessors  2 bzw. 3 Arme  Eingeschlossen: N=30  Ausgewertet: N=29  Drop-Out:  A/B n=1/0 (Aufgrund unabhängiger Erkrankung)  Land: Sydney, Australia,  University of Western Sydney  April 2010 and February 2011 (Erhebung, Rekrutierung von BRCA Patienten) | Brustkrebs  Alter(MW, Range):  (18–70) Jahre  Geschlecht: 100% weiblich  BFI ≥ 4  Chemotherapie mindestens 1 Monat vorher  Diagnose über TCM Stil – welches sich an Symptom Mustern orientiert | **Arm A:**  N=9  Akupunktursitzungen (45 min.) zweimal wöchentlich über 3 Wochen und dann einmal wöchentlich für die letzten 3 Wochen  Alle Nadeln wurden mit einem Park-Gerät für mindestens 20min. eingeführt.  Punkte: bilaterale KI3, KI27, ST36 und SP6 und unilaterale CV4 und CV6 plus bis zu drei Sekundärpunkte, deren Auswahl auf der Differenzialdiagnose des TCM-Paradigmas beruhte  Es wurden Einweg-Vinco-Nadeln aus rostfreiem Stahl (0,25 × 40 mm und 0,22 × 25 mm) verwendet  Die Nadeln wurden gemäß den Standardtexten bis zur Tiefe eingeführt.  Je nach TCM-Diagnose erfolgte eine verstärkende oder reduzierende Stimulation  **Arm B:**  N=10  nichtinvasive Scheinnadel (Stumpf-> keine Hautpenetration) mit dem Park-Gerät an Sham Punkten (Befinden sich am unteren Rücken, am Bauch, am Fuß, am Unterschenkel und am Unterarm)  **ArmC:**  N=10  Wartelistenkontrollgruppe, macht wie gewohnt weiter, wobei der Forscher (BC) alle zwei Wochen Kontakt zu Frauen aufnimmt.  Frauen wurde nach dieser Zeit Akupunktur angeboten. | T0: Baseline, vor der Behandlung (Behandlungsstart)  T1: Nach 2 Wochen  T2: Nach 4 Wochen  T3: Nach 6 Wochen  **Primäre Endpunkte:**  Fatigue:  1. BFI  **Sekundäre Endpunkte:**  Wohlbefinden  2. Wellbeing questionnaire (W-BQ12)  3. Measure Yourself Concerns and Wellbeing questionnaire (MYCaW) | Alle Ergebnisse sind Gruppenunterschiede einer ANOVA  Zu 1.  Mittlerer BFI (SD)  T0:  A: 6.3 (1.7)  B: 6.4 (1.3)  C: 6.5 (0.9)  T1:  A: 3.9 (2.4)  B: 6.1 (1.8)  C: 6.0 (1.9)  Mittlere Differenz (95% CI): 5.3 (4.5 zu 6.2)  P=0.05  T2:  A: 3.1 (2.7)  B: 5.0 (1.8)  C: 5.7 (2.5)  Mittlere Differenz (95% CI): 4.6 (3.6 zu 5.6)  p=0.06  T3:  A: 3.2 (2.4)  B: 4.9 (2.0)  C: 5.4 (1.9)  Mittlere Differenz (95% CI): 4.6 (3.6 zu 5.5)  p=0.08  Zu 2.  Gesamt Wohlbefinden(SD)  T0:  A: 20.7 (4.2)  B: 16.6 (4.7)  C: 20.5 (5.0)  T1:  A: 21.6 (3.1)  B: 17.7 (5.0)  C: 20.9 (4.3)  Mittlere Differenz (95% CI): 19.9 (18.1 zu 21.7)  p=0.13  T2:  A: 22.3 (3.0)  B: 18.0 (5.2)  C: 20.4 (5.4)  Mittlere Differenz (95% CI): 20.2 (18.1 zu 22.1)  p=0.15  T3:  A: 24.4 (4.6)  B: 20.0 (4.8)  C: 22.8 (4.1)  Mittlere Differenz (95% CI): 22.3 (20.3 zu 24.2)  p=0.12  Zu 3.  Mittleres MYCaW Wohlbefinden(SD)  T0:  A: 3.2 (1.5)  B: 3.8 (1.0)  C: 3.4 (1.6)  T1:  A: 2.4 (1.1)  B: 3.7 (1.1)  C: 2.9 (1.6)  Mittlere Differenz (95% CI): 3.0 (2.5 to 3.5)  p=0.14  T2:  A: 2.1 (1.6)  B: 3.3 (0.9)  C: 3.3 (1.4)  Mittlere Differenz (95% CI): 2.9 (2.4 zu 3.4)  p=0.10  T3:  A: 1.6 (1.5)  B: 3.1 (0.8)  C: 3.3 (0.8)  Mittlere Differenz (95% CI): 2.7 (2.1 zu 3.2)  p=0.006  Nach 2 Wochen mittlere Differenz (MD) 5,3, 95 %-KI 4,5 bis 6,2, p = 0,05 und eine signifikante Verbesserung des Wohlbefindens nach 6 Wochen für Akupunktur im Vergleich zur Schein- und Wartelistenkontrolle, MD 2,7, 95 %-KI 2,1 bis 3,2, p=0,006.  A : Alle Teilnehmer verspürten nach der ersten Einführung und noch einmal während der Behandlung das De-Qi-Gefühl.  Die randomisierten Gruppen waren in den meisten Ausgangsmerkmalen vergleichbar, mit Ausnahme von zwei W-BQ12-Scores (20,7 vs. 16,6 vs. 20,5).  Zu Beginn der Studie hatten beide  Gruppen hohe Erwartungen, dass die Akupunktur  bei der Verringerung ihrer Müdigkeit nützlich sein könnte (Akupunktur  100% vs. Placebo 90% p=0,9) | Keine Angaben | Finanzierung durch das Cancer Institute for New South Wales und Nadelspende durch Helio Supply Co. Pty. Ltd.  Autoren geben keinen Interessenskonflikt an | PRO:  Ethikkommission genehmigt (Western Sydney Human Ethics  Committee)  ITT Analyse  Die Analyse untersuchte die demografischen und Ausgangsmerkmale der in die Studie randomisierten Frauen  Ausführliche Interviews, um die Akupunkturerfahrungen der Teilnehmer zu untersuchen und die Auswirkungen und Ergebnisse, die sie möglicherweise erlebt haben, weiter zu untersuchen  STRICA Checkliste  Prüfung der Verblindung  CONTRA:  Keine statistischen Werte für einzelne Gruppenvergleiche  kleine Stichprobengröße (jedoch Pilotstudie)  kurze Beobachtungszeit  6 Akupunkteure – Möglichkeit der unterschiedlichen Behandlung  3 verschiedene Akupunkturpunkte je nach Person möglich – wie wurden diese bei Sham ausgewählt?  Nur blind für 2 von 3 Gruppen  nicht die richtigen Werte für den direkten Vergleich von A und C | 1b |

| Referenz | Studientyp | Patienten-merkmale | Intervention/  Kontrolle, Beobachtung | Untersuchte Endpunkte | Hauptergebnisse | Neben-/ Wechselwir­kungen | Finanzierung/ Interessens-konflikte | Methodische Bemerkungen | Evidenz-klasse (Oxford) |
| --- | --- | --- | --- | --- | --- | --- | --- | --- | --- |
| Molassiotis (2007)  The management of cancer-related fatigue after chemotherapy with acupuncture and acupressure: a randomized controlled trial  Complementary therapies in medicine | RCT  Prospektiv  Monozentrisch  Verblindet für Sham, Hauptermittler  3 Arme  Eingeschlossen: N = 47  Ausgewertet: N=35  Drop-Out:  A/B/C n=2/7/3 (n=0/1/0 in Post verloren, n=1/1/0 gestorben, n=1/0/0 Reisen, n=0/3/1 Krankheit, n=0/0/2 Unbehagen bei der Anwendung der Technik, n=0/2/0 keine Verbesserung)  Land: UK, cancer center  2005 (Erhebung, Rekrutierung von Patienten) | Alter(MW, Range):  53.4 (20-76) Jahre  Geschlecht: 68% weiblich  Aktuell keine Therapie  Krebspatienten, die vor mindestens einem Monat eine Chemotherapie abgeschlossen haben  Patienten mit geplanter Chemotherapie (4 Zyklen EC4P4, gefolgt von 4 Zyklen Paclitaxel als Standardprotokoll).  Patienten mit einem Wert von fünf oder mehr im 10-Punkte-VAS für Fatigue (wobei höhere Punkte einen höheren Ermüdungsgrad anzeigen)  Die häufigsten Diagnosen waren Lymphom und Brustkrebs  Voraussichtliche Überlebenszeit von mehr als 3 Monaten. Kein Plan für eine Chemotherapie, Strahlentherapie oder andere Krebsbehandlungen während des Studienzeitraums. | **Arm A:**  N = 13  Akupunkturen  sechs 20-minütige Sitzungen über 2 Wochen  Drei Punkte (LI4, SP6 und ST36) bilateral genadelt  Tiefe von 0,5–1 Zoll  Von einem erfahrenem Akupunkteur  **Arm B:**  N = 9  Akupressur  täglich für 2 Wochen jeweils 1 Minute lang (Druck auf dieselben Punkte)  Tonisierende Technik (wurde den Patienten beigebracht.  **Arm C:**  N=13  Sham Akupressur  täglich für 2 Wochen in derselben Weise wie Arm B, drei Punkte (LI12, GB33 und BL61), die in der traditionellen chinesischen Medizin nicht auf die gleiche Weise mit „Energie“ verbunden sind | T0: Baseline (Zeitpunkt vor der Randomisierung, ca. 1 Woche)  T1: am Ende der zweiwöchigen Intervention  T2: ca. 2 Wochen nach Ende der Intervention  **Primäre Endpunkte:**  Fatigue:  Multidimensional Fatigue Inventory (MFI)  1. Allgemeine Fatigue  2. Körperliche Fatigue  3. Aktivität  4. Motivation  5. Geistige Fatigue | Mauchlys Sphärizitätstest ergab keine Hinweise darauf, dass die Annahme der Sphärizität verletzt wurde, außer im Fall der Subskala „reduzierte Aktivität“ (p = 0,02).  Der Regressionsansatz (ANCOVA) wurde verwendet  95-prozentige Konfidenzintervalle in T-Tests zwischen zwei Gruppen  1.  Mittelwert (S.D.)  T0:  A: 16.4 (2.4)  B: 16.6 (2.7)  C: 7.8 (2.5)  T1:  A: 10.5 (3.0)  B: 13.4 (3.0)  C: 17.7 (2.6)  T2:  A: 12.8 (3.2)  B: 14 (2.4)  C: 16.9 (3.0)  -> laut Autoren sign. Verbesserung in A und B (ANCOVA zeigt Gruppenunterschiede)  p < 0.001  There was a 36% improvement in fatigue levels in A, while B improved by 19% and C only by 0.6%. | A: 2x punktuelle Blutungen, 1x Bluterguss an einem Punkt, 1x Unwohlsein an einem Punkt (SP6) 1x Übelkeit. B/C: 1x Blutergüsse durch den Druck und Schmerzen in den Punkten nach dem Druck | Unterstützt durch ein Stipendium der European Oncology Nursing Society  (Statistische Unterstützung leistete Dr. Malcolm Campbell) | PRO:  Ethikvotum  ITT principle (primary analyses)  Eingriff gemäß den STRICTA-Empfehlungen beschrieben  Studie wurde vom South Manchester Research & Ethics Committee und dem Ethics Committee der University of Manchester geprüft und genehmigt  Gruppengröße>30 (dennoch zu klein entsprechend der Poweranalyse)  1 Akupunkteur für alle  CONTRA:  Gefördert  Keine direkten Gruppenvergleiche | 1b |

| Referenz | Studientyp | Patienten-merkmale | Intervention/  Kontrolle, Beobachtung | Untersuchte Endpunkte | Hauptergebnisse | Neben-/ Wechselwir­kungen | Finanzierung/ Interessens-konflikte | Methodische Bemerkungen | Evidenz-klasse (Oxford) |
| --- | --- | --- | --- | --- | --- | --- | --- | --- | --- |
| Lu (2012)  The feasibility and effects of acupuncture on quality of life scores during chemotherapy in ovarian cancer: Results from a pilot, randomized sham-controlled trial  Medical Acupuncture | RCT  Prospektiv  Bizentrisch  Blind für alle Beteiligten, außer behandelnden Akupunkteur  2 Arme  Eingeschlossen: N=21  Ausgewertet: N=15 für Endpunkt 2 und n=14 für Endpunkt 1  Drop-Out:  A/B n=3/3 (n=4 Krankheitsprogression, n=1 Nebenwirkungen der Chemotherapie, n=0/1 G-CSF benutzt)  Land: USA  Weitere Angaben in Vorgängerstudie | Alter(MW, Range):  A//B: 50.8 –/+ 10.6// 50 –/+ 9.9  Geschlecht: 100% weiblich  Frauen mit primärem Eierstockkrebs; primärer Peritonealkrebs; papillärer seröser Gebärmutterkrebs; und gemischten mesodermale Tumoren der Gebärmutter, des Eierstocks oder des Eileiters, die sich einer standardmäßigen myelosuppressiven Chemotherapie (hauptsächlich intravenös [i.v.] Carboplatin und Paclitaxel in einem 21-Tage-Zyklus) unterzogen.  Keine Verwendung von Granulozyten-Kolonie-stimulierendem Faktor (G-CSF) während der Behandlung.  Keine Vorgeschichte einer symptomatischen Herzerkrankung und unkontrollierte schwere psychiatrische Störungen (schwere Depressionen/Psychosen) | **Arm A:**  N=8  10 Sitzungen Akupunkturbehandlung, 2–3-mal pro Woche, für 30 min., beginnend 1 Woche vor Zyklus 2 der Chemotherapie und endend vor Zyklus 3 der Chemotherapie, ein Zeitraum von 4 Wochen.  Manuelle und Elektrostimulation. An neun Akupunkturpunkte (17 Nadelungsstellen).  De Qi Gefühl manuell einmal an mindestens zwei Punkten an den Beinen jedes Teilnehmers und an einem Punkt an den Armen erzeugt. Zusätzlich wurde ein Elektroakupunkturstimulator (EA) beidseitig mit einer Frequenz von 20–25 Hz an die Beine des Patienten angeschlossen, deren Intensität der Stimulation langsam erhöht wurde, bis die Patienten „oral“ berichteten, dass sie das Gefühl verspürten.  **Arm B:**  N=7  Sham-Akupunktur.  Gleiches Zeitschema wie bei A  5 Nichtakupunktur  Punkte (9 Nadelungsstellen), lagen in der Nähe von Verumpunkten, jedoch außerhalb der Meridiane. Sobald die Nadeln minimal eingeführt waren, waren keine Handmanipulationen und kein De Qi mehr erlaubt. Es wurde ein identischer, aber deaktivierter EA-Stimulator verwendet. | T0: Baseline (Vor Akupunkturstart)  T1: Nach den Akupunktursitzungen (ca. 4 Wochen nach T0)  **Primäre Endpunkte:**  Lebensqualität  1. Cancer-Quality-of-Life Questionnaire-Core 30 Item (EORTC-QLQ-C30)  2. Quality of Life Questionnaire–Ovarian Cancer Module-28 Item (QLQ-OV28) | Zu 1.:  Nach Adjustierung für Baseline-Werte, nur sign. Unterschiede für Skala SF (social function) zugunsten A (p=0.03), keine sign. Unterschiede für Global Lebensqualität (Mittelwert, SD) zu  T1:  A(n=7): 69.0 (22.4)  B(n=7): 63.1 (17.3)  p= 0.94  oder  Fatigue zu  T1:  A(n=7): 34.9 (18.6)  B(n=7): 36.5 (8.4)  p= 0.89  Eine mittlere Score-Änderung von >=10 im QLQ-C30 wurde als klinisch bedeutsame moderate Veränderung interpretiert  Ein p-Wert < 0,05 wurde als statistisch signifikant angesehen  Vergleiche der Ausgangsmittelwerte wurden mit dem Mann-Whitney-U-Test sowie einem t-Test für unabhängige Stichproben durchgeführt  Die EORTC-QLQ-C30-Subscores wurden im Akupunkturarm verbessert, einschließlich der mittleren Scores für soziale Funktion (SF), Schmerzen und Schlaflosigkeit (p = 0,05). Allerdings war nach Bereinigung um Ausgangsunterschiede nur der SF-Score im Arm mit aktiver Akupunktur signifikant höher als im Arm mit Scheinakupunktur (p = 0,03). | Es wurden keine signifikanten unerwünschten Ereignisse im Zusammenhang mit der Akupunkturnadelung beobachtet | Autoren geben keine konkurrierenden finanziellen Interessen an | PRO:  Es wurden Anstrengungen unternommen, um die gleiche  Kontaktzeit zwischen den behandelnden Akupunkteuren und den  und Patienten in den beiden Studienarmen zu gewährleisten.  Eine visuelle Barriere wurde verwendet, um jedem Patienten die Sicht auf die Nadelungsstellen zu versperren.  Verblindungssicherung durch validierte Glaubwürdigkeitsskala ausgefüllt von Patienten und gleiche Kontaktdauer von Akupunkteuren mit Patienten  CONTRA:  Drop-out 29% (unklar durch unterschiedliche viele Ergebnisse)  Keine ITT Analyse  kleine Stichprobengröße  kurze Beobachtungszeit | 1b |

| Referenz | Studientyp | Patienten-merkmale | Intervention/  Kontrolle, Beobachtung | Untersuchte Endpunkte | Hauptergebnisse | Neben-/ Wechselwir­kungen | Finanzierung/ Interessens-konflikte | Methodische Bemerkungen | Evidenz-klasse (Oxford) |
| --- | --- | --- | --- | --- | --- | --- | --- | --- | --- |
| Deng (2018)  Acupuncture for reduction of symptom burden in multiple myeloma patients undergoing autologous hematopoietic stem cell transplantation: a randomized sham-controlled trial  Supportive care in cancer | RCT  Prospektiv  Monozentrisch  Blind für alle Beteiligten (Probanden und Gutachter, außer behandelnden Akupunkteur  2 Arme  Eingeschlossen: N=63  Ausgewertet: N=60  Drop-Out:  A/B n=2/1, keine Gründe gegeben, in A vor Intervention, in B nach einer Sitzung  Land: USA, Memorial Sloan Kettering (MSK) Cancer Center  May 2013 and January 2016 | Alter(MW, Range):  A/B: 59 (54, 66)/ 58 (55, 64)  Geschlecht: 63,33% weiblich  Erwachsene Patienten mit multiplem Myelom, die sich einer hochdosierten Melphalan-Behandlung mit anschließender autologer HCT (AHCT) am MSK unterziehen sollten.  Ausschlusskriterien waren eine absolute Neutrophilenzahl (ANC) von weniger als 200/μl, eine Thrombozytenzahl von weniger als 20.000/μl und eine Akupunkturbehandlung in den letzten 4 Wochen vor Tag 1. | **Arm A:**  N=29  Echte Akupunktur.  Akupunkturbehandlung 20min. einmal täglich für 5 Tage, beginnend am Tag nach der Stammzelleninfusion und einen Tag vor dem Erhalt einer konditionierenden Chemotherapie  Behandlungsplan wie für alle anderen Patienten am MSK.  Akupunkturpunkte: GV20, Ex-HN3, HT7, PC6, ST36, SP6, KI3, LR3 und Ear Shen Men. Akupunkturnadeln: 36 Gauge × 40 mm Länge und 40 Gauge × 30 mm Länge, hergestellt von Seirin Corporation  **Arm B:**  N=31  Sham-Akupunktur.  Die Behandlungen erfolgten in beiden Armen nach dem gleichen Zeitplan.  Identisch mit ArmA, außer dass keine Nadel in die Haut eingeführt wird  Der Akupunkteur befestigte ein leeres Akupunkturnadelführungsrohr aus Kunststoff am Knochenbereich neben jedem Akupunkturpunkt, um ein wahrnehmbares Gefühl zu erzeugen, und klebte dann eine Nadel 20 Minuten lang mit einem Stück Klebeband auf die Hautoberfläche | T0: Baseline (1 bis 2 Wochen vor der konditionierenden Chemotherapie)  T1: täglich von Tag 2 bis Tag 5 nach Stammzellinfusion (Tag 0, 1 Tag vor Akupunkturstart)  T2: 15 Tage nach der Transplantation.  T3: 30 Tage nach der Transplantation.  **Primäre Endpunkte:**  Symptombelastung  1. MD Anderson Symptom Inventory (MDASI) | (ANCOVA), angepasst an den MDASI-Ausgangswert und die Art der Chemotherapie (stationär vs. ambulant), da diese zur Stratifizierung der Randomisierung verwendet wurden zur Vergleichbarkeitsbeurteilung  95 %- Konfidenzintervalle (95 %-KIs) zu jedem Zeitpunkt und ermittelten P-Werte durch den t-Test ermittelt um die MDASI-Werte in den Akupunkturgruppen mit der historischen Kontrolle in einer Post-hoc-Analyse zu vergleichen.  P < 0. 05 stat. relevant  Mehr Schmerzmitteleinnahmen bei B (Odds Ratio 5,31, P = 0,017).  1.:  MDASI Symptomwert,  Mittelwert (SD)  Tage 0 bis + 5 (T0, T1):  A: 1.23 (1.24)  B: 1.66 (1.14)  Differenz: − 0.19  95% KI: 0.60 bis 0.23  p= 0.4  T2:  A 1.34 (1.49)  B: 2.10 (1.52)  Differenz: − 0.47  95% KI: − 1.03 to 0.09  p= 0.1  T3:  A: 1.15 (1.10)  B: 1.67 (1.40)  Differenz: − 0.37  95% CI: − 0.92 to 0.18  p=0.2  Es gibt keine Fatigue spezifischen Werte  A vs. B nicht sign. für gesamten MDASI-Kernsymptomwerte und Symptominterferenzwerte während der Transplantation  (p= 0,4 bzw. 0,3)  2.: A vs. B  Für die Gesamtwerte der MDASI-Kernsymptome und der Symptom-Interferenz-Scores: (p = 0,10 bzw. 0,3).  Bei der Verringerung von Übelkeit, Appetitlosigkeit und Schläfrigkeit signifikant wirksamer (p = 0,042, 0,025 bzw. 0,010)  Bei der Reduzierung von Übelkeit, Appetitlosigkeit und Schläfrigkeit signifikant effektiver(p = 0,042, 0,025 bzw. 0,010).  3.: A vs. B  Für die MDASI-Kernsymptom-Gesamtwerte und die Symptom-Interferenz-Scores: (p = 0,2 und 0,4) | Akupunktur wurde gut vertragen. Es gab keine signifikanten Unterschiede in der Häufigkeit unerwünschter Ereignisse im Zusammenhang mit der Akupunktur zwischen den beiden Gruppen | Die Studie wurde durch einen Zuschuss des Gateway for Cancer Research und den MSK Integrative Medicine and Translational Research Grant finanziert. Auch unterstützt vom National Institutes of Health/National Cancer Institute (NIH/NCI) Cancer Center Support Grant P30  CA008748, der AC Israel Foundation und dem Byrne Fund.  Die Geldgeber spielten keine Rolle bei der Konzeption und Durchführung der Studie; Sammlung, Verwaltung, Analyse und Interpretation der Daten; Vorbereitung, Überprüfung oder Genehmigung des Manuskripts; und Entscheidung, das Manuskript zur Veröffentlichung einzureichen | PRO:  Ethikvotum  In beiden Gruppen waren die Augen der Patienten mit Pflastern abgedeckt, sodass sie den Behandlungsvorgang nicht sehen konnten  Die Wirksamkeit der Verblindung wurde beurteilt, indem die Patienten gebeten wurden, zu erraten, welcher Gruppe sie zugeordnet wurden.  Bewertung des Medikamentengebrauchs vom Ausgangswert bis zum 5. Tag  Die Glaubwürdigkeitswerte waren in jeder Gruppe sehr ähnlich (P > 0,9), was darauf hindeutet, dass die Verblindung beibehalten wurde  Poweranalyse  CONTRA:  Keine ITT Analyse  Kaum Baselineinformationen  Keine Werte für Skalen außerhalb der Signifikanten | 1b |

| Referenz | Studientyp | Patienten-merkmale | Intervention/  Kontrolle, Beobachtung | Untersuchte Endpunkte | Hauptergebnisse | Neben-/ Wechselwir­kungen | Finanzierung/ Interessens-konflikte | Methodische Bemerkungen | Evidenz-klasse (Oxford) |
| --- | --- | --- | --- | --- | --- | --- | --- | --- | --- |
| Hou (2017):  Transcutaneous electrical acupoint stimulation (TEAS) relieved cancer-related fatigue in non-small cell lung cancer (NSCLC) patients after chemotherapy, Journal of thoracic disease | RCT  Prospektiv  Monozentrisch  Verblindet (Pat., Krankenpfleger*innen); keine Informationen bzgl. der Forscher  3 Arme  Eingeschlossen: N=169  Ausgewertet:  N=162  Drop-out: A/B/C n = 2/3/2 (zu krank, um das Programm fortzusetzen, oder sie konnten eine Chemotherapie nicht vertragen)  Land : Shanghai  oncology ward of a specialist pulmonary hospital  Juli 2014 bis Juli 2015 | NSCLC, Gemcitabin + Platinbasierte Chemotherapie  Alter(Bereich):  20-80  Geschlecht: 75,4% weiblich | **Arm A:**  N=57  Transcutaneous electrical nerve stimulation TEAS.  AkupunkturpunkteQihai (CV6), Keshu (UB17) und Zusanli (ST36).  Dichtdisperse Frequenz von 30/100 Hz und eine Intensität von 6–15 V  Sitzungen von 30 Minuten an den Tagen 1, 2, 3, 5, 8, 11, 14, 28.  Für TEAS wurde G6805-II verwendet mit dichtdisperser Frequenz von 30/100 Hz und einer Intensität von 6–15 V.  Die optimale Intensität wurde so angepasst, dass ein leichtes Zucken des regionalen Muskels entsprechend der individuellen maximalen Toleranz verbleibt  **Arm B:**  N=49  Sham TEAS  Verfahren und Dauer der TEAS-Behandlung waren dieselben wie in ArmA, die Behandlung wurde jedoch an nahegelegenen Stellen außerhalb der Akupunkturpunkte angewendet  **Arm C:**  N=56  Kontrollgruppe  erhielten routinemäßige Pflege im Hinblick auf eine strikte Beobachtung der Zustandsveränderungen, Ernährungspflege, Schutz vor Venenembolien und die routinemäßige Verwendung von Antiemetika und gastroprotektiven Mitteln. | T0: Baseline (Tag vor der Chemotherapie)  T1: Tag 8  T2: Tag 28  **Primäre Endpunkte:**  CNI  1. Revised Piper Fatigue Scale (RPFS)  **Sekundäre Endpunkte:**  Demografischer Status | Zu1.:  T1:  A: 2.85±1.62  B: 2.59±1.18  C: 2.68±1.36  F-Test: 0.488  p = 0.615  T2:  Signifikanter Unterschied zwischen den 3 Gruppen:  A: 2.06±0.90  B: 2.80±1.34  C: 3.00±1.29  F-Test: 9.784  p = <0.01  Alle Dimensionen zeigten statistisch signifikante Unterschiede, mit Ausnahme der sensorischen Ermüdung (P = 0,50). Allerdings hatte A (Mittelwert = 2,48) einen niedrigeren Wert für sensorische Ermüdung als C oder B (MW = 3,20 und MW = 2,72).  Mehrfacher Vergleich:  A vs. B:  Verhalten f.: p=0.919  Affektiv f.: p=0.927  Sensorisch f.: p=0.122  Kognitiv f.: p=0.795  A vs. C:  Verhalten f.: p=0.001  Affektiv f.: p=0.002  Sensorisch f.: p=0.016  Kognitiv f.: p= <0.01  B vs. C:  Behavioral f.: p=0.031  Affektiv f.: p=0.001  Sensorisch f.: p=0.428  Kognitiv f.: p=0.016 | Bei insgesamt 57 Patienten der TEAS-Gruppe traten keine unerwünschten Reaktionen auf. | Die Studie wurde durch ein Stipendium der National Nature and Science Foundation of China unterstützt  Laut Autoren kein Einfluss dadurch | PRO:  Gruppen zur Baseline vergleichbar  Ethikvotum (ethics committee of the Shanghai Pulmonary Hospital)  Es gab keinen signifikanten Unterschied in den Ausgangsmerkmalen zwischen den Studienabbrechern und denen, die die Studie abgeschlossen hatten (P > 0,05).  Poweranalyse  CONTRA:  Die täglichen Aktivitäten der Teilnehmer könnten sich auf ihren Müdigkeitsgrad ausgewirkt haben  Eine nur dreimalige Datenerhebung innerhalb von 28 Tagen könnte die Merkfähigkeit verstärken, was sich auf die Ergebnisse auswirken könnte    Keine ITT  Keine genauen Angaben zur Verblindung oder Nebenwirkungen  Es wurden nicht alle ursprünglich im Protokoll angegebenen Akupunkturpunkte tatsächlich in der Studie verwendet | 1b |

| Referenz | Studientyp | Patienten-merkmale | Intervention/  Kontrolle, Beobachtung | Untersuchte Endpunkte | Hauptergebnisse | Neben-/ Wechselwir­kungen | Finanzierung/ Interessens-konflikte | Methodische Bemerkungen | Evidenz-klasse (Oxford) |
| --- | --- | --- | --- | --- | --- | --- | --- | --- | --- |
| Mao (2014):  Electroacupuncture for fatigue, sleep, and psychological distress in breast cancer patients with aromatase inhibitor-related arthralgia: a randomized trial  Journal article | RCT  Prospektiv  Monozentrisch  Verblindet für Sham (Pat.)  3 Arme  Eingeschlossen: N=76  Randomisiert:  N=67  N bis T4:  N=59  Drop-out: n = 9 (7: Schmerzen, die unter den Einschlusskriterien lagen, 1: hatte starke Schmerzen, die nichts mit AIs zu tun hatten, 1: wollte nicht teilnehmen)  Vier (6 %) und acht (12 %) Patienten aller randomisierten Patienten konnten vor Woche 8 bzw. 12 nicht mehr nachuntersucht werden.  Land: USA Abramson Cancer Center of the Hospital of the University of Pennsylvania September 2009 bis Mai 2012 | Alter(MW, Range):  59.7, 41-76  Geschlecht: 100% weiblich  Frauen mit einer Vorgeschichte von Brustkrebs im Frühstadium (Stadium I-III), die derzeit einen Aromatasehemmer (Anastrozol, Letrozol oder Exemestan) erhielten, seit mindestens drei Monaten Gelenkschmerzen hatten, die sie auf ihre AI zurückführten, und mit Schmerzen von mindestens vier oder mehr auf einer numerischen Bewertungsskala mit 11 Punkten (0–10) in der vorangegangenen Woche, Angabe von mindestens 15 Tagen mit Schmerzen in den vorangegangenen 30 Tagen.  Ausgeschlossen: Teilnehmer mit metastasiertem Brustkrebs (Stadium IV) oder einer Blutungsstörung in der Vergangenheit. | **Arm A:**  N=22  Electro-Akupunktur  Sitzungen: zwei Wochen lang zweimal pro Woche, dann sechs weitere Wochen lang wöchentlich, also insgesamt zehn Behandlungen über acht Wochen.  Nadeln: 30 mm oder 40mm und 0.25 Gauge wurden eingeführt, bis „De Qi“ (Wundegefühl, Kribbeln usw.) vom Patienten gemeldet wurde.  Zwei Elektrodenpaare wurden an den Nadeln neben dem/den schmerzenden Gelenk(en) mit einer Zwei-Hertz-Elektrostimulation verbunden, die von einem TENS-Gerät bereitgestellt wurde. Die Nadeln wurden 30 Minuten lang an Ort und Stelle belassen und zu Beginn und am Ende der Therapie kurz manipuliert.  **Arm B:**  N=22  Sham Akupunktur;  Die Häufigkeit und Dauer der Behandlungen zwischen der EA- und der SA-Gruppe waren identisch.  Nicht durchdringende Streitberger-Nadeln an Nicht-Akupunktur- und Nicht-Triggerpunkten, mindestens 5 cm vom Gelenk entfernt, wo der Schmerz als maximal empfunden wurde. Die Akupunkteure vermieden es, die „De Qi“-Empfindungen hervorzurufen, indem sie die Nadeln, abgesehen von ihrem ersten Kontakt mit der Haut, nur minimal manipulierten. Anstatt den Nadeln einen kleinen elektrischen Strom zuzuführen, stellten die Akupunkteure den Drehknopf des TENS-Geräts auf einen anderen Kanal, sodass die Testperson das Blinken des Lichts beobachten konnte, ohne Strom zu erhalten  **Arm C:**  N=23  Kontrollgruppe mit normaler Pflege auf der Warteliste | T0: Baseline  T1: Woche 2  T2: Woche 4  T3: Woche 8  T4: Woche 12 (vier Wochen nach der Behandlung)  **Primäre Endpunkte:**  Schmerzintensität und Interferenz  1. Brief Pain Inventory (BPI)  **Sekundäre Endpunkte:**  Fatigue  2. Brief Fatigue Inventory (BFI)  Schlaf  3. global score of the Pittsburgh Sleep Quality Index (PSQI)  Psychische Belastung  4. Hospital Anxiety and Depression Scale (HADS) | Zu2.:  Sign. Verbesserung in A im Vergleich zu C über alle Zeitpunkte hinweg (gemischtes Modell; p=0.0095), mit sign. Unterschieden zu T3 und T4;  Änderung von T0 (Mittelwert, 95% CI):  T2:  A: −0.4 (−1.6 to 0.7)  B: −0.5 (−1.7 to 0.7)  C: −0.1 (−0.8 to 0.6)  A vs. C:  −0.3 (−1.6 to 1.0, p=0.57);  B vs C:  −0.4 (−1.8 to 1.0, p=0.38)  T3:  A: −1.4 (−2.7 to −0.1)  B: −0.6 (−1.7 to 0.5)  C: 0.5 (−0.2 to 1.3)  A vs. C:  −2.0 (−3.4 to −0.5, p=0.0034); Cohen *d*=0.96  B vs C:  −1.2 (−2.5 to 0.1, p=0.046)  T4:  A: −1.4 (−2.7 zu −0.1)  B: −0.7 (−1.6 zu 0.1)  C: 0.2 (−0.8 zu 1.2)  A vs. C:  −1.6 (−3.2 zu −0.07, p=0.022); Cohen *d*=0.86  B vs C:  −0.9 (−2.2 zu 0.3, p=0.091) | In den Studien wurden nur wenige geringfügige und selbstlimitierende Nebenwirkungen (z. B. stechende Schmerzen, lokale Blutergüsse) festgestellt | NIH/NCCAM R21 AT004695; Dr. Mao ist Träger des NCCAM K23 AT004112-Preises | PRO:  ITT zwischen dem EA- und dem WLC-Arm sowie zwischen dem SA- und dem WLC-Arm basierend auf Interaktionen zwischen Zeit und Intervention in den Mixed-Effects-Modellen  Gruppen zu Baseline vergleichbar  Das Institutional Review Board der University of Pennsylvania genehmigte das Studienprotokoll.  Die Verblindung wurde anhand der Glaubwürdigkeitsbewertung in Woche 8 bewertet. Die Teilnehmer betrachteten sowohl EA als auch SA als glaubwürdig (4.3 vs. 4.0, p=0.54))  Poweranalyse  CONTRA:  Unter den Teilnehmern erhielten 21 (95,4 %) in der EA-Gruppe und 20 (90,5 %) in der SA-Gruppe alle zehn Behandlungen  A vs. B nicht berichtet  Evtl. Bias: Die Patienten der WLC-Gruppe durften nach 12 Wochen Nachbeobachtungszeit 10 echte Akupunkturbehandlungen erhalten.  Zwei zugelassene, nichtärztliche Akupunkteure mit 8 und 20 Jahren Erfahrung -> sehr unterschiedlich | 1b |
